# Transcriptome analysis reveals a lncRNA-miRNA-mRNA regulatory network in OsRpp30-mediated disease resistance in rice

**DOI:** 10.1186/s12864-023-09748-w

**Published:** 2023-10-26

**Authors:** Minghua Li, Wei Li, Meixia Zhao, Zhiqiang Li, Guo-Liang Wang, Wende Liu, Chun Liang

**Affiliations:** 1 Department of Biology, Miami University, Oxford, OH 45056 USA; 2 Department of Plant Pathology, Ohio State University, Columbus, OH 43210 USA; 3 Department of Microbiology and Cell Science, University of Florida, Gainesville, FL 32611 USA; 4 State Key Laboratory for Biology of Plant Diseases and Insect Pests, Institute of Plant Protection, Chinese Academy of Agricultural Sciences, Beijing, 100193 China

**Keywords:** OsRpp30, mRNA, lncRNA, miRNA, ceRNA, Disease resistance, Rice

## Abstract

**Background:**

Long non-coding RNAs (lncRNAs) play critical roles in various biological processes in plants. Extensive studies utilizing high-throughput RNA sequencing have revealed that many lncRNAs are involved in plant disease resistance. *Oryza sativa* RNase P protein 30 (*OsRpp30*) has been identified as a positive regulator of rice immunity against fungal and bacterial pathogens. Nevertheless, the specific functions of lncRNAs in relation to OsRpp30-mediated disease resistance in rice remain elusive.

**Results:**

We conducted a comprehensive analysis of lncRNAs, miRNAs, and mRNAs expression patterns in wild type (WT), *OsRpp30* overexpression (OsRpp30-OE), and *OsRpp30* knockout (OsRpp30-KO) rice plants. In total, we identified 91 differentially expressed lncRNAs (DElncRNAs), 1671 differentially expressed mRNAs (DEmRNAs), and 41 differentially expressed miRNAs (DEmiRNAs) across the different rice lines. To gain further insights, we investigated the interaction between DElncRNAs and DEmRNAs, leading to the discovery of 10 *trans-* and 27 *cis-*targeting pairs specific to the OsRpp30-OE and OsRpp30-KO samples. In addition, we constructed a competing endogenous RNA (ceRNA) network comprising differentially expressed lncRNAs, miRNAs, and mRNAs to elucidate their intricate interplay in rice disease resistance. The ceRNA network analysis uncovered a set of gene targets regulated by lncRNAs and miRNAs, which were found to be involved in pathogen recognition, hormone pathways, transcription factor activation, and other biological processes related to plant immunity.

**Conclusions:**

Our study provides a comprehensive expression profiling of lncRNAs, miRNAs, and mRNAs in a collection of defense mutants in rice. To decipher the putative functional significance of lncRNAs, we constructed *trans-* and *cis-*targeting networks involving differentially expressed lncRNAs and mRNAs, as well as a ceRNA network incorporating differentially expressed lncRNAs, miRNAs, and mRNAs. Together, the findings from this study provide compelling evidence supporting the pivotal roles of lncRNAs in OsRpp30-mediated disease resistance in rice.

**Supplementary Information:**

The online version contains supplementary material available at 10.1186/s12864-023-09748-w.

## Background

Rice (*Oryza sativa*) is an important staple crop that serves as a primary food source for over half of the global population. However, the persistent threat of pathogen attacks, ranging from viruses, bacteria, to fungi, poses a significant challenge, leading to reduced yields and jeopardized food safety [1]. Extensive research conducted over the past three decades has shed light on the mechanisms underlying rice immunity in response to pathogen infections, resulting in the identification of numerous genes encoding resistance receptors, signaling molecules, and defense-related proteins [2–4]. Additionally, small RNAs, including microRNAs (miRNAs), have been characterized as playing essential roles in rice immunity [5–7]. In contrast, the function of lncRNAs and their relationship with miRNAs and their target transcripts in rice defense responses remain largely unexplored.

lncRNAs are a class of RNAs that are longer than 200 nucleotides (nts) and lack protein-coding potential. While they do not undergo translation to produce proteins, they exhibit significant similarities with messenger RNAs (mRNAs). Similar to mRNAs, the majority of lncRNAs are transcribed by RNA polymerase II and undergo post-transcriptional modification, such as 5’ capping, alternative splicing, and 3’ poly A addition [8]. Unlike mRNAs, lncRNAs tend to be less evolutionarily conserved, with lower but more pronounced tissue- and condition-specific expression patterns [9]. lncRNAs were thought to be by-products of transcriptional noise. However, more and more evidence has demonstrated that they play critical roles in diverse biological processes, exerting gene regulation at multiple levels, including histone modification, transcriptional regulation, and post-transcriptional regulation [10]. lncRNAs are often categorized into different subtypes based on their genomic locations in relation to protein-coding genes. Among these, long intergenic non-coding RNAs (lincRNAs) were the first discovered lncRNAs because of their widespread transcription in the regions that do not encode proteins [11].

Several well-characterized lncRNAs have been identified in plants, such as cold-assisted intronic noncoding RNA (*COLDAIR*), cold-induced long antisense intragenic RNA (*COOLAIR*), and long-day-specific male-fertility-associated RNA (*LDMAR*) [12–14]. *COOLAIR* and *COLDAIR* mediate the flowering process in *Arabidopsis thaliana* (*A. thaliana*), while *LDMAR* regulates photoperiod-sensitive male sterility in rice. Additionally, a leaf-expressed lncRNA named *Xanthomonas oryzae* pv. *oryzae* (*Xoo*) induced lncRNA 1 (*ALEX1*) has been found to enhance rice resistance to bacterial blight by activating the jasmonate (JA) signaling pathway [15]. In contrast to plants, numerous lncRNAs have been implicated in innate immunity in humans or mice, such as long intergenic noncoding RNA erythroid prosurvival (*lincRNA-EPS*), *lnc13*, and *Mirt2* [16–19]. Despite the identification of numerous plant lncRNAs facilitated by RNA sequencing (RNA-Seq) techniques in recent decades, the functions of the majority of these lncRNAs remain elusive.

One of the primary roles of lncRNAs is their ability to regulate gene expression either in *cis* or *trans*. lncRNAs can regulate the expression of their neighboring protein-coding genes in *cis* through direct interactions with regulatory proteins or binding to specific motifs on the DNA sequence of the nearby target genes. A notable example is the lncRNA known as homeobox A (HOXA) transcript at the distal tip (*HOTTIP*). Positioned in proximity to the HOXA genes cluster, *HOTTIP* regulates the expression of HOXA genes in *cis* [20]. *HOTTIP* directly binds to the adaptor protein WD repeat-containing protein 5 (WDR5) and recruits the WDR5-myeloid/lymphoid or mixed-lineage leukemia (MLL) histone methyltransferase complexes to the HOXA locus. This recruitment leads to the deposition of histone H3 lysine 4 trimethylation (H3K4me3) on the promoter regions of HOXA genes, ultimately activating their transcription. Conversely, certain lncRNAs, such as TCF21 antisense RNA inducing demethylation (*TARID*), can form an RNA-DNA-DNA triplex (R-loop) with its nearby gene *TCF21* on the promoter region [21]. Consequently, the growth arrest and DNA damage inducible-alpha protein (GADD45A) recognizes the R-loop and recruits the ten-eleven translocation 1 (TET1) protein to facilitate the demethylation of the DNA on the promoter region of *TCF21*, leading to increased transcription of *TCF21*.

In addition to their *cis*-regulatory functions, lncRNAs also regulate the expression of distant genes in *trans* through mechanisms such as chromatin interaction or post-transcriptional regulation. In *A. thaliana*, the lncRNA auxin-regulated promoter loop (*APOLO*) activates the expression of *trans* targets via an R-loop-induced regulation [22]. Upon auxin stimulation, the *APOLO* transcript is expressed and inserted into the promoter region of its *trans* targets through sequence complementarity, forming R-loops. These R-loops disrupt the binding of polycomb factors, such as heterochromatin protein 1 (LHP1), resulting in the deposition of the repressive mark histone H3 lysine 27 trimethylation (H3K27me3) and subsequent activation of the target genes. In addition to regulating gene expression through chromatin interaction, lncRNA plays a vital role in the post-transcriptional regulation of their *trans* targets. For instance, the lncRNA terminal differentiation-induced ncRNA (*TINCR*) can bind to the 25-nt ‘TINCR box’ motifs within target mRNAs, leading to the recruitment of Staufen homolog 1 (STAU1) and subsequent stabilization of the mRNAs [23].

MicroRNAs are typically ~ 21–22 nts long, single-strand RNAs that play important roles in diverse biological processes [24]. Through sequence complementarity, miRNAs can interact with target mRNAs, wherein the complementary sequences are referred to as miRNA response elements (MREs). Depending on the degree of complementarity between miRNAs and their target mRNAs, miRNAs can induce mRNA decay or translational repression of the target mRNAs [25]. lncRNAs containing MREs can function as competing endogenous RNAs (ceRNAs) or miRNA sponges, thereby reducing the availability of miRNAs for target mRNAs and regulating their expression. For instance, in tomato, *lncRNA39026* acts as a ceRNA by sequestering miR168a, leading to the up-regulation of the resistance gene solanum lycopersicum argonaute1 (*SIAGO1*) and enhanced resistance against *Phytophthora infestans* infection [26].

Accumulating evidence has indicated that lncRNAs are involved in the response of rice to various pathogens. For instance, a transcriptome analysis on rice black-streaked dwarf virus (RBSDV) infected rice plants identified 1273 lncRNAs, with 22 showing differential expression in response to RBSDV infection [27]. In another study focused on rice lncRNAs during *Magnaporthe oryzae* (*M. oryzae*) infection, 161 lncRNAs were found to be differentially expressed following pathogen inoculation [28]. Furthermore, a comprehensive examination revealed 567 rice lncRNAs with distinct expression patterns in response to *Xoo* [15].

OsRpp30 is a subunit of Ribonuclease P (RNase P), an enzyme complex primarily responsible for the cleavage and maturation of tRNAs [29–31]. Our previous studies have demonstrated that *OsRpp30* enhances disease resistance against *M. oryzae* and *Xoo* in rice [32]. However, whether lncRNAs and miRNAs are involved in OsRpp30-mediated broad-spectrum disease resistance in rice remains unclear. Here we performed an integrated analysis of the transcriptomic profile of lncRNA, miRNA, and mRNA in wild type (WT), *OsRpp30* overexpression (OsRpp30-OE), and *OsRpp30* knockout (OsRpp30-KO) rice plants to elucidate the regulatory interactions among lncRNAs, miRNAs, and mRNAs that contribute to the improved plant immunity conferred by *OsRpp30* overexpression. In this study, a total of 91 DElncRNAs, 1671 DEmRNAs, and 41 DEmiRNAs were identified across the three rice lines. Particularly, 10 *trans-* and 27 *cis-*targeting pairs of DElncRNA-DEmRNAs were identified. Moreover, miRNAs from four families were found to be involved in the lncRNA-miRNA-mRNA ceRNA regulatory network. Together, these findings unveil a complex lncRNA-miRNA-mRNA regulatory network that likely plays an important role in OsRpp30-mediated disease resistance in rice.

## Results

### Identification and characterization of novel lncRNAs in rice

**Fig. 1:**
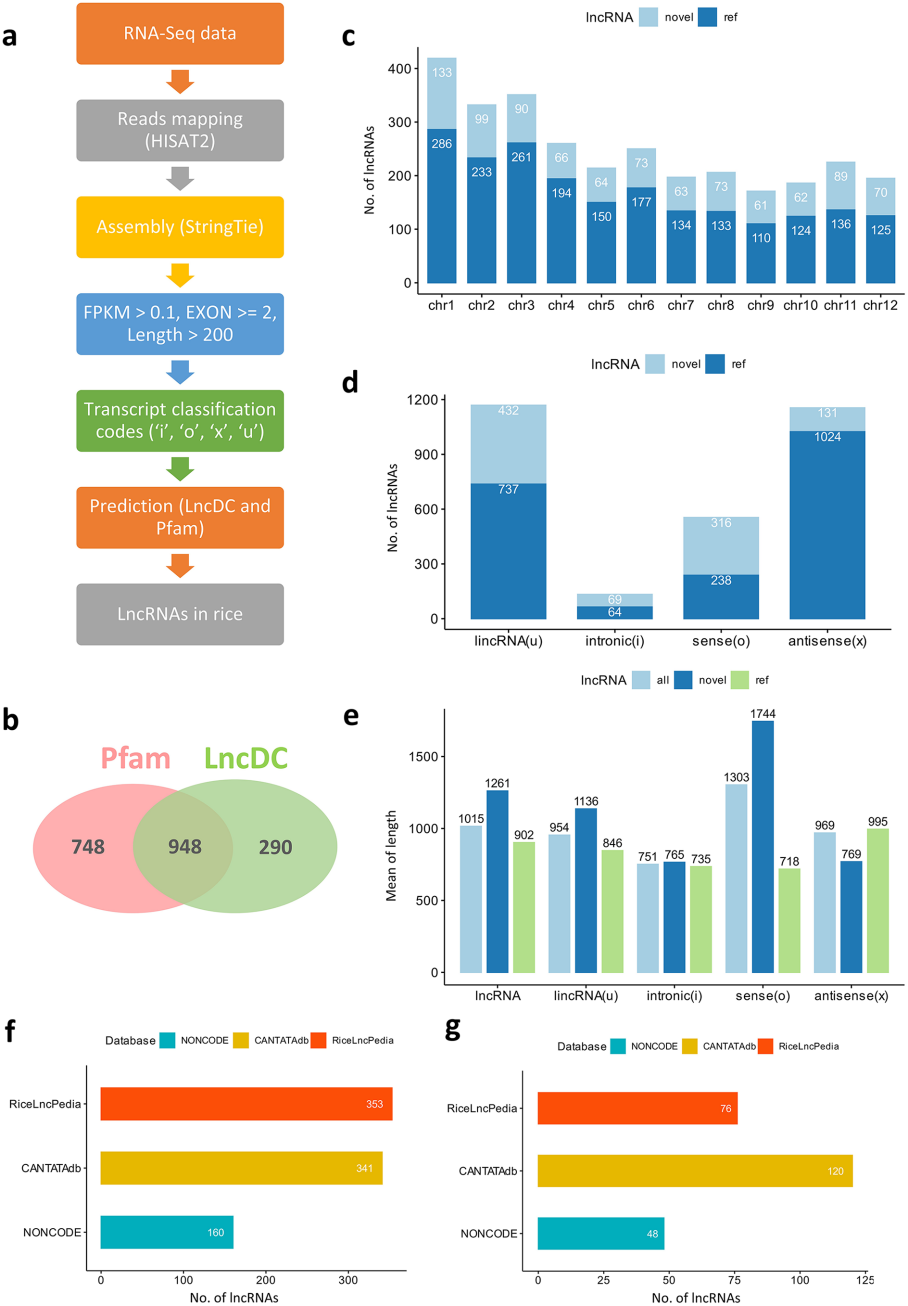
Identification and characterization of rice lncRNAs. **a** The pipeline for the prediction of novel lncRNAs in rice. **b** The number of novel lncRNAs predicted by both Pfam and LncDC. **c** The number of predicted novel (*novel*) and annotated reference (*ref*) lncRNAs in different rice chromosomes. **d** The number of predicted novel and annotated reference lncRNAs from different categories. **e** The average length of different types of lncRNAs for all rice lncRNAs (*all*), predicted novel lncRNAs, and annotated reference lncRNAs. **f** The number of predicted novel lncRNAs with > = 90% identity and > = 100 nt alignment length against lncRNAs in various rice lncRNA databases. **g** The number of predicted novel lncRNAs with > = 90% identity and > = 90% length coverage rate against lncRNAs from different rice lncRNA databases

We employed a pipeline, as depicted in Fig. 1a, to identify novel lncRNAs in rice. On average, approximately 72 million sequencing read pairs were obtained from each rice sample, with a mapping rate of 94% to the reference genome (See Supplementary Table S1, Additional File 1). Overall, 948 highly confident novel lncRNA transcripts from 794 lncRNA gene loci were predicted in congruence by both LncDC [33] and Pfam [34] programs (Fig. 1b and Additional File 2).

The numbers of lncRNAs varied across different chromosomes, with the largest number (419) on chromosome 1 and the smallest number (171) on chromosome 9, likely due to the variation of chromosomal length (Fig. 1c). The numbers of intronic lncRNAs (code ‘i’) and sense lncRNAs (code ‘o’) were similar between the newly identified (novel) and reference lncRNAs. Among the intronic lncRNAs, 69 were newly identified and 64 were reference lncRNAs. Similarly, we found 316 novel and 238 reference sense lncRNAs. There were more long intergenic non-coding RNAs (lincRNA, classification code ‘u’) from the reference lncRNAs (737) compared to the novel lncRNAs (432). Additionally, we noticed that there were many more reference antisense lncRNAs (code ‘x’, 1024) than the novel antisense lncRNAs in our dataset (Fig. 1d) (131). In general, both the novel lncRNAs we identified and the reference lncRNAs were found in intergenic regions or overlapping with genes on the opposite strand.

We also examined the length distribution of different types of lncRNAs in rice (Fig. 1e). The average length of the novel lncRNAs we identified was 1261 nts, which was significantly longer than the average length of reference lncRNAs (902 nts, p-value = 1.16 × 10^− 24^, two-tailed Student’s *t*-test). This longer average length of novel lncRNAs is likely due to the fact that we removed the low-confidence single exon transcripts during the prediction process. Among the different categories of lncRNAs, intronic lncRNAs were the shortest on average, as they are located within introns and cannot exceed the length of the corresponding intron. In contrast, sense lncRNAs were generally longer than other types of lncRNAs, particularly among the predicted novel lncRNAs. Both lincRNAs and antisense lncRNAs had similar average lengths although they were shorter than sense lncRNAs.

Next, we examined whether these novel lncRNAs we identified were present in other rice lncRNA databases, such as NONCODE, CANTATAdb, and RiceLncPedia [35–37]. We found that 160, 341, and 353 lncRNAs showed alignment hits with identities of 90% or higher and alignment lengths of 100 nts or longer in NONCODE, CANTATAdb, and RiceLncPedia, respectively (Fig. 1f). These aligned lncRNAs shared conserved sequences with the lncRNAs found in the databases, suggesting that they likely belong to the same lncRNA family. We further focused on the predicted novel lncRNAs that not only had a 90% identity but also exhibited a high coverage rate when aligned with the lncRNAs from the databases. Specifically, we considered those lncRNAs where at least 90% of the sequences showed 90% or higher identity with the lncRNAs in the databases. In total, we found 48, 120, and 76 predicted novel lncRNAs that displayed high similarity to the lncRNAs in the NONCODE, CANTATAdb, and RiceLncPedia databases, respectively (Fig. 1g and Additional File 3).

Furthermore, we then observed that 47 of the predicted novel lncRNAs showed similarity to the lncRNAs present in both the NONCODE and CANTATAdb databases. Similarly, eight lncRNAs were confirmed by both RiceLncPedia and NONCODE, and 22 lncRNAs were confirmed by both RiceLncPedia and CANTATAdb. Remarkably, a total of eight predicted novel lncRNAs exhibited high similarity to lncRNAs in all three rice lncRNA databases. These eight lncRNAs are MSTRG.10686.3, MSTRG.12637.1, MSTRG.13742.1, MSTRG.16892.1, MSTRG.19604.1, MSTRG.24992.1, MSTRG.25244.1, and MSTRG.8373.1 (See Supplementary Table S2, Additional File 1).

### Differential expression analysis reveals potential roles of lncRNAs in rice disease resistance mediated by OsRpp30

**Fig. 2:**
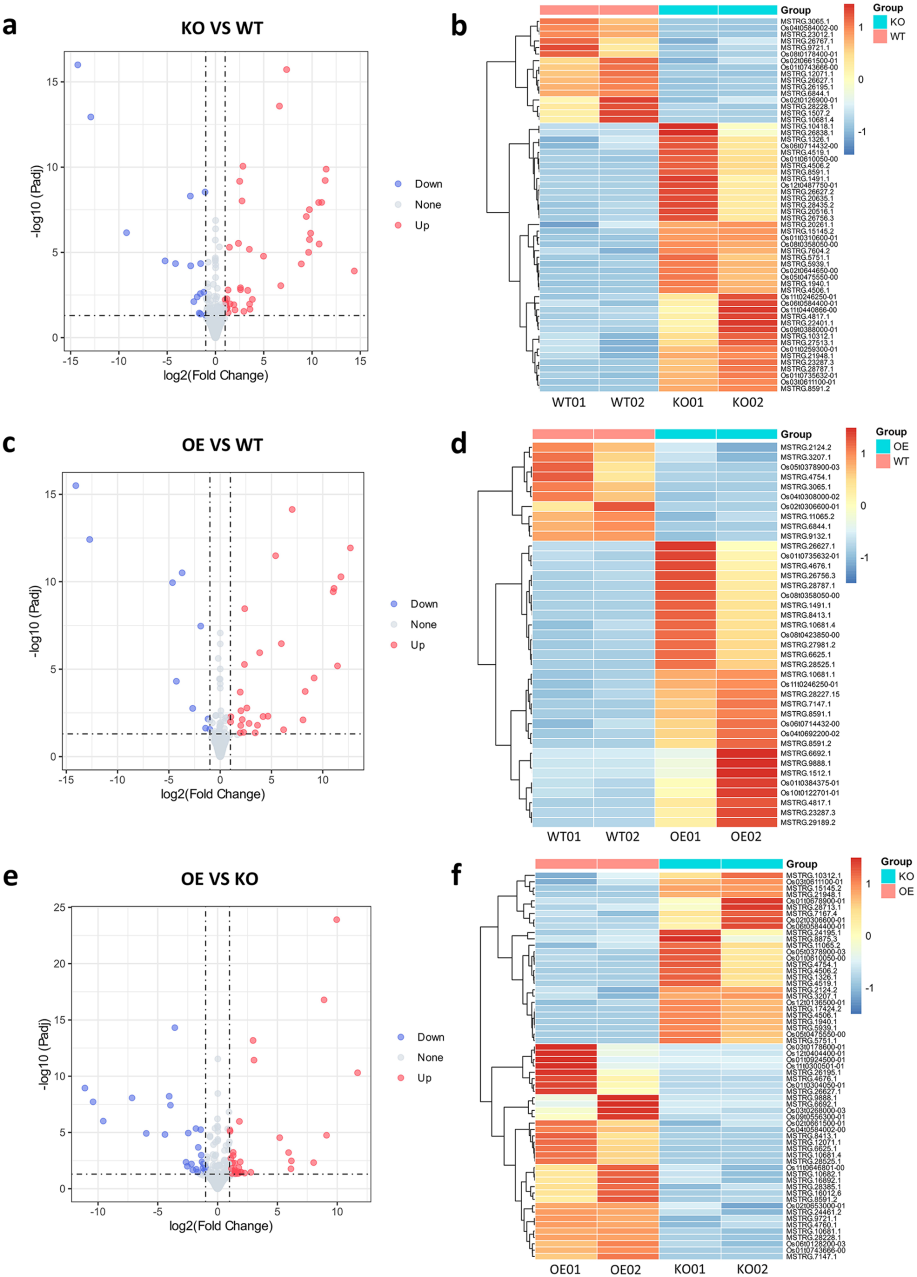
Differential expression of lncRNAs between individual rice lines. **a** The volcano plot of DElncRNAs between OsRpp30-KO and WT. Red dots represent up-regulated lncRNAs and blue dots represent down-regulated lncRNAs with statistical significance (|log2(FC)| > 1, p-adjusted < 0.05). **b** The heat map of DElncRNAs between OsRpp30-KO and WT. The color from blue to red represents the expression level from low to high. **c** The volcano plot of DElncRNAs between OsRpp30-OE and WT. **d** The heat map of DElncRNAs between OsRpp30-OE and WT. **e** The volcano plot of DElncRNAs between OsRpp30-OE and OsRpp30-KO. **f** The heat map of DElncRNAs between OsRpp30-OE and OsRpp30-KO.

The RNA expression profiles of lncRNAs and mRNAs from different rice lines were analyzed using principal component analysis (PCA). The results revealed that the samples from the same rice line were clustered together, indicating that the replicates of the same genotype exhibited similar RNA expression profiles (See Supplementary Figure S1a, Additional File 1). We further compared the expression levels of lncRNAs among the samples from the three lines, WT, OsRpp30-KO (susceptible), and OsRpp30-OE (resistant). A total of 57 DElncRNAs, including 41 up-regulated and 16 down-regulated lncRNAs, were identified between OsRpp30-KO and WT plants (Fig. 2a and Additional File 4). To visualize the expression pattern of these DElncRNAs between OsRpp30-KO and WT samples, we normalized the expression of DElncRNAs using Fragments Per Kilobase of transcript per Million mapped reads (FPKM) and generated a heat map (Fig. 2b). Notably, the lncRNA MSTRG.10312.1 exhibited high expression in OsRpp30-KO samples (average FPKM of 17) but was less expressed in WT samples (average FPKM of 6). This sense lncRNA is located on the reverse strand of chromosome 2: 7,323,916-7,325,579 and partially overlaps with the protein-coding gene *Os02g0230300* on the same strand.

In addition, we identified 29 up-regulated and 10 down-regulated lncRNAs between OsRpp30-OE and WT samples (Fig. 2c and Additional File 4). One interesting newly identified lncRNA, MSTRG.4676.1, was found to be a sense lncRNA located on the forward strand of chromosome 10: 11,079,933 − 11,087,253. MSTRG.4676.1 displayed minimal expression in WT samples but was highly expressed in OsRpp30-OE samples (average FPKM of 3.6) (Fig. 2d). Interestingly, MSTRG.4676.1 overlapped with the reference lncRNA transcript *Os10t0360600-01*, sharing similar exons, suggesting that MSTRG.4676.1 may represent a novel transcript isoform of *Os10t0360600-01*. Notably, although MSTRG.4676.1 displayed differential expression, no expression of *Os10t0360600-01* was detected in any of the samples.

Next, we compared the expression of lncRNAs between OsRpp30-OE and OsRpp30-KO samples and identified 61 DElncRNAs, including 34 up-regulated and 27 down-regulated in OsRpp30-OE samples (Fig. 2e and f and Additional File 4). Of particular interest was *Os03t0268000-03*, a reference lncRNA derived from the protein phosphatase 1(*OsPP1*) gene, which also has two other protein-coding isoforms, *Os03t0268000-01* and *Os03t0268000-02*. *Os03t0268000-03* exhibited high expression in OsRpp30-OE samples (average FPKM of 4.2), while its expression was lower in OsRpp30-KO samples (average FPKM of 0.87). In addition, we discovered *Os12t0136500-01*, a reference lincRNA that displayed up-regulation in OsRpp30-KO samples with an average FPKM of 117.3. Despite being down-regulated in the OsRpp30-OE samples, it maintained a relatively high average FPKM of 20.75, indicating that it may play an important biological role in rice.

In addition to the pairwise comparisons between OsRpp30-KO and WT, between OsRpp30-OE and WT, and between OsRpp30-OE and OsRpp30-KO, we also investigated lncRNAs with specific differential expression patterns in each line. Given that OsRpp30-OE exhibited the highest resistance while OsRpp30-KO displayed the highest susceptibility, we focused on the lncRNAs that display differential expression when comparing OsRpp30-KO samples to both OsRpp30-OE and WT samples (KO vs. OE&WT), as well as OsRpp30-OE samples to both OsRpp30-KO and WT samples (OE vs. KO&WT).

**Fig. 3:**
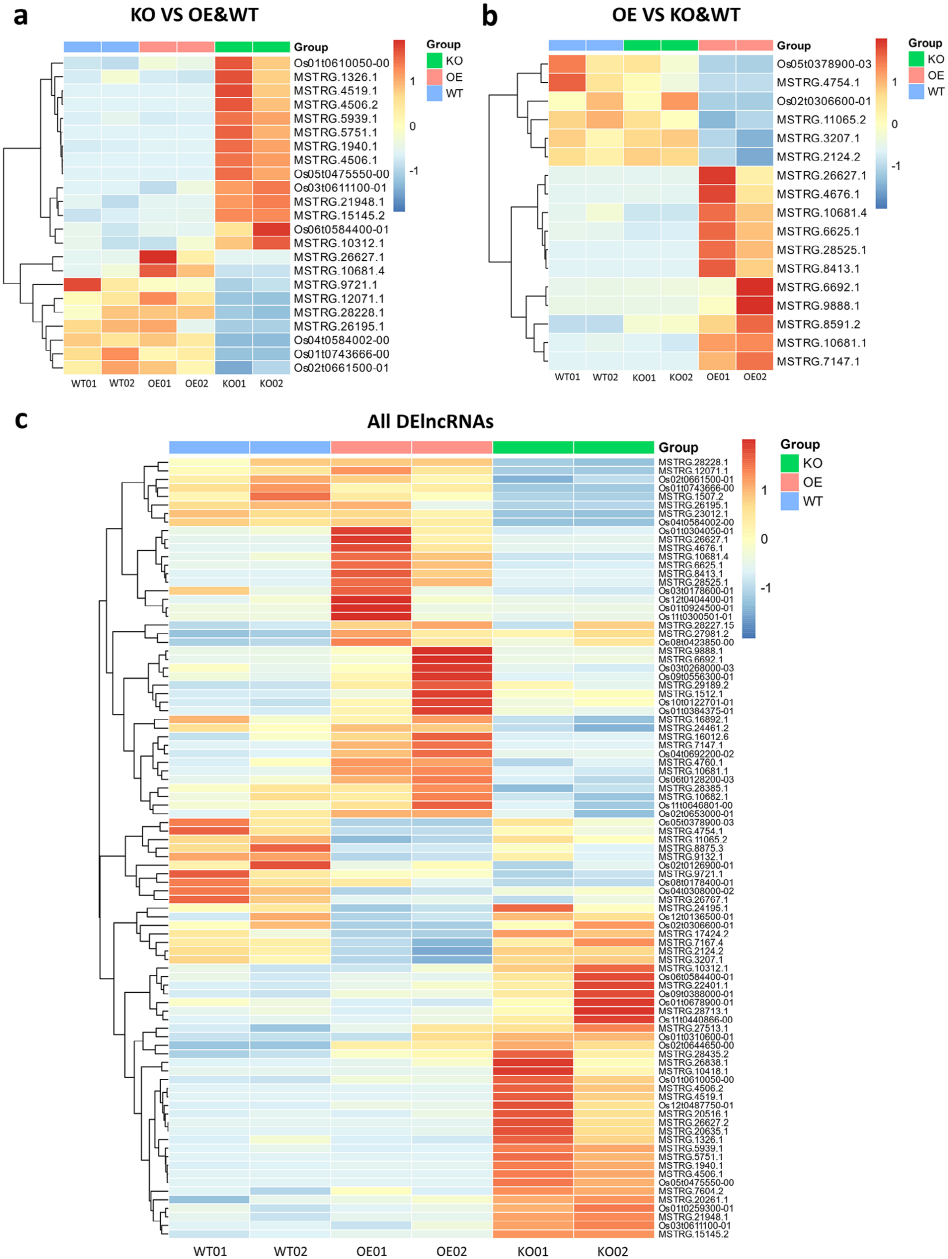
Differential expression of lncRNAs in all three rice lines. **a** The heat map of DElncRNAs between OsRpp30-KO and both OsRpp30-OE and WT (OE&WT) samples. **b** The heat map of DElncRNAs between OsRpp30-OE and both OsRpp30-KO and WT (KO&WT) samples. **c** The heat map of all DElncRNAs among all three lines without including the DElncRNAs between WT and both OsRpp30-OE and OsRpp30-KO (OE&KO) samples

The expression levels of DElncRNAs from the comparison between KO and OE&WT are shown in Fig. 3a. We identified 14 up-regulated lncRNAs in OsRpp30-KO samples but down-regulated in OE&WT samples, with the majority of them belonging to lincRNAs (9). Conversely, nine lncRNAs were down-regulated in OsRpp30-KO samples but up-regulated in OE&WT samples. Additionally, we examined the expression levels of DElncRNAs from the comparison between OE and KO&WT (Fig. 3b). The results showed that 11 lncRNAs were up-regulated in OsRpp30-OE samples, including five lincRNAs and six sense lncRNAs. Only six lncRNAs were down-regulated in OsRpp30-OE samples compared to KO&WT samples, with four being lincRNAs. Lastly, we extracted DElncRNAs differentially expressed in at least two different rice lines, specifically, OsRpp30-KO vs. WT, OsRpp30-OE vs. WT, and OsRpp30-OE vs. OsRpp30-KO. In total, we identified 91 DElncRNAs that are likely crucial for OsRpp30-mediated disease resistance in rice (Fig. 3c).

Notably, we discovered several DElncRNAs that bear resemblance to the disease-responsive lncRNAs documented in other studies. For instance, TCONS_00004581 is a rice lncRNA down-regulated in response to *Xoo* infection, indicating a negative association with rice immunity [15]. Interestingly, we observed that MSTRG.1326.1 overlapped with TCONS_00004581 on the same strand and shared identical start positions. Sequence alignment revealed that they possess a 100% identity with 78% coverage, suggesting that they are likely isoforms derived from the same lncRNA gene locus. Moreover, similar to TCONS_00004581, MSTRG.1326.1 was down-regulated in OsRpp30-OE samples, further supporting its negative relationship with rice immunity. Another *Xoo*-responsive lncRNA, TCONS_00490059, is known to be down-regulated after *Xoo* infection [15]. MSTRG.15145.2 overlapped with TCONS_00490059 with 99.56% identity and 95% coverage. Corresponding to the negative association of TCONS_00490059 with rice immunity, MSTRG.15145.2 showed down-regulation in OsRpp30-OE samples.

### Differential expression of mRNAs reveals insights into OsRpp30-mediated disease resistance in rice

**Fig. 4:**
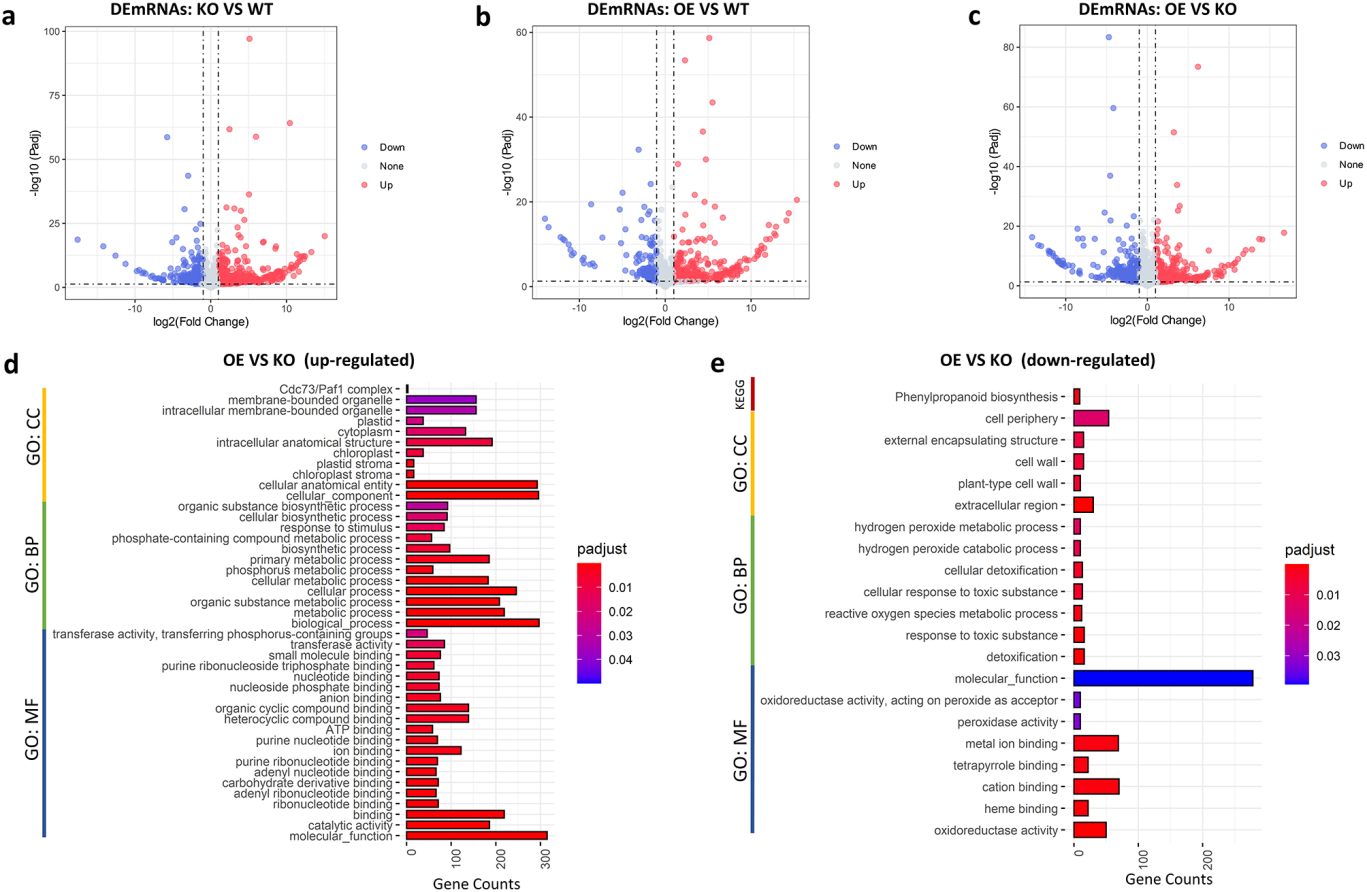
Differential expression of mRNAs between individual rice lines. **a** The volcano plot of DEmRNAs between OsRpp30-KO and WT. Red dots represent up-regulated mRNAs and blue dots represent down-regulated mRNAs with statistical significance (|log2(FC)| > 1, p-adjusted < 0.05). **b** The volcano plot of DEmRNAs between OsRpp30-OE and WT. **c** The volcano plot of DEmRNAs between OsRpp30-OE and OsRpp30-KO. **d** The Gene ontology (GO) terms enriched for up-regulated mRNAs between OsRpp30-OE and OsRpp30-KO. **e** The GO terms and KEGG pathways enriched for down-regulated mRNAs between OsRpp30-OE and OsRpp30-KO.

We next performed mRNA expression analysis to investigate the differential expression patterns associated with OsRpp30-mediated disease resistance. Between OsRpp30-KO and WT samples, we identified 670 up-regulated and 377 down-regulated mRNAs, as depicted in Fig. 4a and Additional File 5. Similarly, in comparison between OsRpp30-OE and WT samples, we found 571 DEmRNAs, with 400 mRNAs up-regulated and 171 mRNAs down-regulated (Fig. 4b and Additional File 5). Additionally, we identified 1018 DEmRNAs between OsRpp30-OE and OsRpp30-KO samples, consisting of 523 up-regulated and 495 down-regulated mRNAs (Fig. 4c and Additional File 5). In total, we identified 1671 DEmRNAs, which are likely to play crucial roles in OsRpp30-mediated disease resistance in rice.

Our analysis revealed a substantial number of DEmRNAs associated with plant immunity. Notably, several genes including *Pita*, *HDT701*, *Os11g0644700*, *Os06g0707350*, *Os01g0350300*, *Os05g0143550*, *Os08g0543500*, *OsCrRLK1L14* and *Oschib1* were up-regulated in OsRpp30-OE but down-regulated in OsRpp30-KO. Among these genes, *Pita*, *Os06g0707350*, and *Os08g0543500* encode proteins belonging to the nucleotide-binding site leucine-rich repeat (NBS-LRR) class, which are crucial for recognizing pathogen effectors and activating general immune responses [4]. *Pita*, in particular, is a rice blast resistance gene against *M. oryzae* [38, 39]. Additionally, we have identified two more NBS-LRR protein-coding genes, *Os11gRGA5* [40] and *BPH26* [41], which exhibited elevated expression levels in OsRpp30-OE samples compared to WT samples. Moreover, *Os11g0644700*, *Os01g0350300*, and *OsCrRLK1L14* encode plant disease resistance response proteins, while *Os05g0143550* encodes a protein similar to the blast and wounding-induced mitogen-activated protein kinase. *Oschib1* produces chitinase, a pathogenesis-related protein involved in rice defense against fungal pathogens. Interestingly, we observed the up-regulation of *HDT701* in OsRpp30-OE samples compared to OsRpp30-KO. *HDT701* is a histone H4 deacetylase, known as a negative regulator in rice innate immunity, and directly interacts with OsRpp30 [32, 42].

In our analysis, we observed reduced expressions of key resistance-related genes in OsRpp30-KO samples, such as *OsCPS2*, *WAK112*, *Os09g0481600*, and *HAP2E*, compared to WT samples. *OsCPS2* is an *ent*- copalyl diphosphate (CPP) synthase essential for the biosynthesis of natural antibiotic products, phytoalexins. Studies have shown that overexpression of *OsCPS2* enhances rice defense against both fungal and bacterial pathogens [43]. *WAK112*, a wall-associated kinase, is known to be involved in rice blast resistance [44]. *HAP2E*, a rice heme activator protein (HAP) gene, is associated with rice resistance to pathogens and abiotic stress [45].

In contrast, we observed significantly elevated expression levels of *OsERF6*, *OsERF922*, *Os01g0112800*, *GH3-2*, *OsMPK20-1*, *Os11gRGA3*, *OsCrRLK1L16*, and *Os07g0130100* in OsRpp30-KO samples compared to WT samples. Of particular interest, *OsERF922*, an apetela2/ethylene response factor (AP2/ERF) type transcription factor, functions as a negative regulator of rice defense against blast pathogens [46].

Furthermore, *OsPR1-101*, *Os10g0398100*, *GLP8-3*, *GLP8-4*, *Os03g0126700*, *Os05g0440100*, *Oschib2* and *CHT2* were up-regulated in OsRpp30-KO samples but down-regulated in OsRpp30-OE samples. Notably, while *Oschib1*, *Oschib2*, and *CHT2* are all chitinases, we observed contrasting expression profiles between *Oschib1* and *Oschib2*/*CHT2*, suggesting potential functional divergence or regulation between these closely related genes involved in rice defense mechanisms.

In light of our previous findings demonstrating the direct interaction between *OsRpp30* and the rice histone deacetylase *HDT701*, we aimed to identify any differentially expressed mRNAs involved in histone modification in rice [32]. Notably, we found that some histone-modifying writers or erasers were differentially expressed, including *HDT701*, *Os05g0440100*, *Os02g0180400*, *JMJ716*, *OsSET11*, *OsSET18*, *OsSET30*, and *SDG714*. Among these genes, *Os02g0180400* is a GCN5-related N-acetyltransferase (GNAT) domain-containing protein with similarity to histone H4 acetyltransferase. *HDT701* and *Os05g0440100* are histone deacetylases. Interestingly, our data indicated that *HDT701* was up-regulated in OsRpp30-OE samples, while *Os05g0440100* and *Os02g0180400* were up-regulated in OsRpp30-KO samples. *OsSET11*, *OsSET18*, *OsSET30*, and *SDG714* are su(var)3–9, enhancer of zeste, and trithorax (SET) domain-containing methyltransferases. In particular, *OsSET11* showed significantly higher expression in OsRpp30-OE samples than in both WT and OsRpp30-KO samples, whereas *OsSET18* exhibited elevated expression in OsRpp30-OE samples compared to WT samples. Conversely, *OsSET30* displayed reduced expression levels in OsRpp30-KO samples compared to WT samples. Additionally, we identified *JMJ716*, which shares similarities with a jumonji C (JmjC) domain-containing protein exhibiting histone demethylase activity [47]. Both *JMJ716* and *SDG714* were up-regulated in OsRpp30-KO samples. Together, several histone modification-related protein-coding genes were differentially expressed in our samples, suggesting their potential roles in OsRpp30-mediated disease resistance in rice.

To gain insights into the putative functions of the DEmRNAs, we performed Gene Ontology (GO) and Kyoto Encyclopedia of Genes and Genomes (KEGG) pathway enrichment analyses. Many GO terms were enriched for the protein-coding genes up-regulated in OsRpp30-OE samples (OsRpp30-OE vs. OsRpp30-KO) (Fig. 4d). In terms of the biological process category, GO terms related to stimulus-response, phosphorus metabolic, and phosphate-containing compound metabolic process were enriched, suggesting a role of OsRpp30 in rice disease response. GO terms underlying molecular functions included transferase activity, nucleotide binding, ribonucleotide binding, organic/heterocyclic compound binding, ATP binding, ion binding, and catalytic activity were enriched, all of which are closely associated with plant immunity. On the other hand, a few GO terms and one KEGG pathway were enriched for the down-regulated mRNAs in OsRpp30-OE samples (OsRpp30-OE vs. OsRpp30-KO) (Fig. 4e). Notably, GO biological processes, such as the hydrogen peroxide metabolic process, cellular detoxification, cellular response to toxic substances, and reactive oxygen species metabolic process, as well as GO molecular functions, including oxidoreductase activity, peroxidase activity, tetrapyrrole binding, and oxidoreductase activity were enriched in these down-regulated mRNAs.

We also performed GO and KEGG pathway analyses for DEmRNAs between OsRpp30-OE and WT (See Supplementary Figures S2a and S2b, Additional File 1) and between OsRpp30-KO and WT samples (See Supplementary Figures S2c and S2d, Additional File 1). We found that some stress response-associated GO biological processes were enriched for the down-regulated mRNAs in OsRpp30-KO samples compared to WT samples (OsRpp30-KO vs. WT), including response to stress, response to stimulus, response to abiotic stimulus, terpenoid metabolic process, terpenoid biosynthetic process, diterpenoid biosynthetic process, and others.

### Distinct miRNA expression profiles across OsRpp30-associated samples

**Fig. 5:**
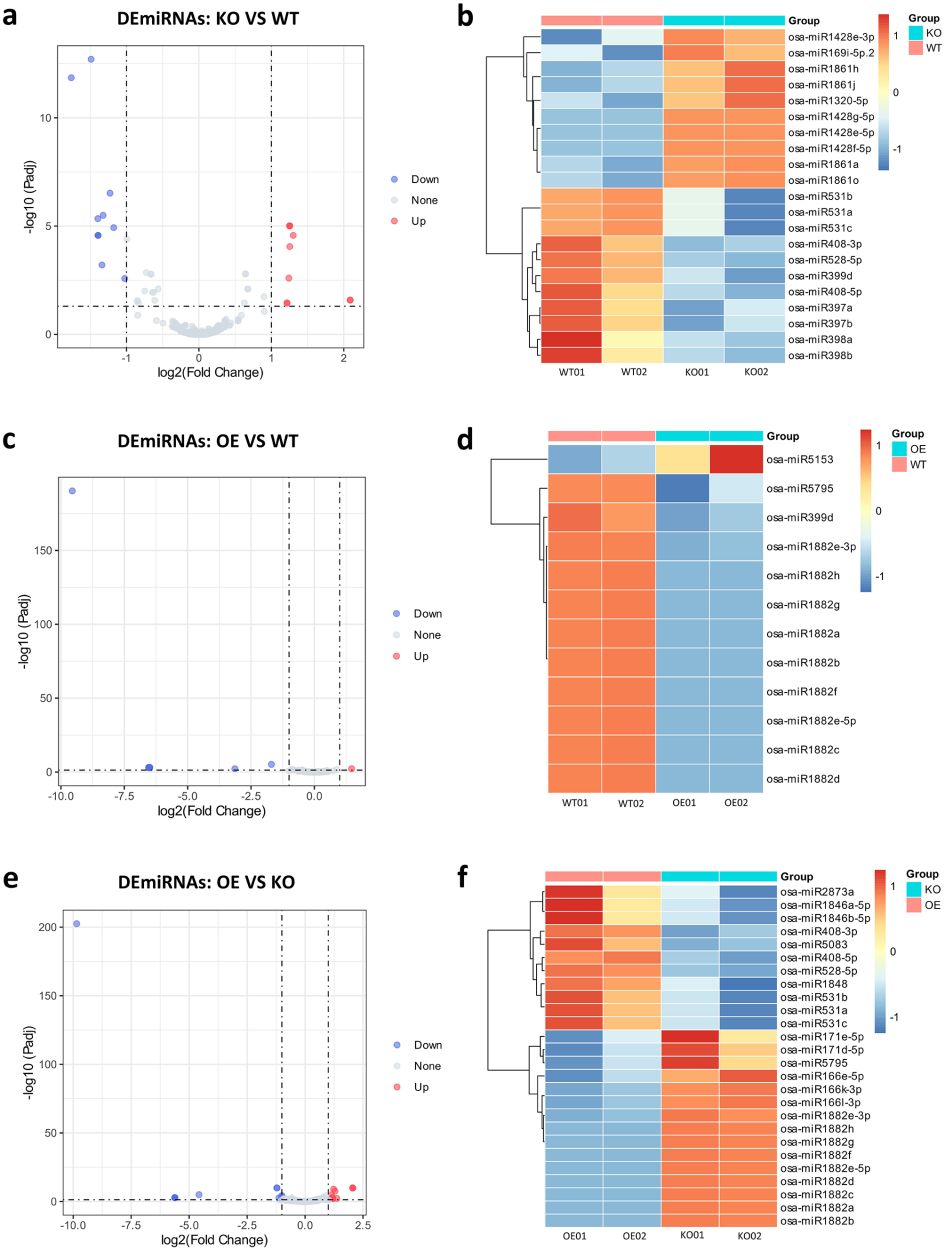
Differential expression of miRNAs between individual rice lines. **a** The volcano plot of DEmiRNAs between OsRpp30-KO and WT. Red dots represent up-regulated miRNAs and blue dots represent down-regulated miRNAs with statistical significance (|log2(FC)| > 1, p-adjusted < 0.05). **b** The heat map of DEmiRNAs between OsRpp30-KO and WT. The color from blue to red represents the expression level from low to high. **c** The volcano plot of DEmiRNAs between OsRpp30-OE and WT. **d** The heat map of DEmiRNAs between OsRpp30-OE and WT. **e** The volcano plot of DEmiRNAs between OsRpp30-OE and OsRpp30-KO. **f** The heat map of DEmiRNAs between OsRpp30-OE and OsRpp30-KO.

To identify miRNAs involved in the regulation of differentially expressed lncRNAs and mRNAs in response to fungal and bacterial pathogens, we sequenced small RNAs from the same rice samples (See Supplementary Table S1, Additional File 1). The miRNA expression levels of the samples from the same rice lines were clustered together, indicating similar miRNA expression profiles among replicates of the same genotype (See Supplementary Figure S1b, Additional File 1). We identified a total of 10 up-regulated and 11 down-regulated miRNAs between OsRpp30-KO and WT samples (Fig. 5a and b, and Additional File 6). Notably, the *osa-miR1428* family miRNAs were up-regulated in OsRpp30-KO samples. Interestingly, the lncRNA *Os03t0611100-01*, which serves as a precursor of *miR1428e* and *miR1428d*, was also up-regulated in OsRpp30-KO samples. This finding further supports the notion that *Os03t0611100-01* is the precursor of the *miR1428* family miRNAs. Previous research has demonstrated that *osa-miR169* acts as a negative regulator in rice immunity against *M. oryzae* by inhibiting nuclear factor Y-A (NF-YA) genes [6]. Our data showed that *osa-miR169i-5p.2* was up-regulated in OsRpp30-KO samples, suggesting that it plays a critical role in OsRpp30-mediated disease resistance in rice. Interestingly, *miR1320* has been implicated in *miR164*-mediated immunity enhancement in rice [48]. However, in our study, we found the up-regulation of *osa-miR1320-5p* in OsRpp30-KO samples, while *miR164* did not show differential expression. Additionally, *miR398b* has been identified as a positive regulator in rice defense against rice blast, leading to increased H_2_O_2_ levels after *M. oryzae* attack [7]. In our study, *miR398b* exhibited reduced expression in OsRpp30-KO samples compared to WT samples.

Only one up-regulated and 11 down-regulated miRNAs were identified between OsRpp30-OE and WT samples (Fig. 5c and d, and Additional File 6). Moreover, we identified 11 up-regulated and 15 down-regulated miRNAs between OsRpp30-OE and OsRpp30-KO samples (Fig. 5e and f, and Additional File 6). One of note is *miR1846*, which acts as a negative regulator of 1-aminocyclopropane-1-carboxylic acid oxidase (ACO), an enzyme involved in ethylene (ET) biosynthesis [49]. ET has a dual function in plant immunity, as it can either promote disease or enhance resistance [50]. In our study, we observed the up-regulation of *osa-miR1846a-5p* and *osa-miR1846b-5p* in OsRpp30-OE samples, while their mRNA target *ACO4* was down-regulated. This finding suggests a potential mechanism for OsRpp30-mediated disease resistance in rice by suppressing ET synthesis. Previous studies have shown that *miR528* negatively regulates rice resistance against viruses [51, 52]. In our study, *osa-miR528-5p* showed significantly higher expression in both OsRpp30-OE and WT samples compared to OsRpp30-KO samples. The *miR171* family, including *osa-miR171*, is highly conserved and plays an essential role in stress responses, such as salt and drought tolerance, as well as pathogen resistance in rice [53–55]. In particular, the expression level of *osa-miR171* in rice increases after sheath blight disease pathogen infection [56]. Our data revealed the down-regulation of *osa-miR171e-5p* and *osa-miR171d-5p* in OsRpp30-OE samples. Additionally, *osa-miR166e-5p*, *osa-miR166k-3p*, and *osa-miR166l-3p* were down-regulated in OsRpp30-OE samples. The *osa-miR166* family miRNAs are known to participate in stress response in rice. For instance, under salt conditions and mineral deficiencies, *osa-miR166k-3p* and *osa-miR166l-3p* are down-regulated in both panicles and shoots [57]. Moreover, *osa-miR166* family miRNAs have been implicated in rice resistance against fungal and viral pathogens, including rice blast fungus, rice dwarf virus, and rice stripe virus [58, 59].

Next, we examined the expression levels of miRNAs between OsRpp30-KO and OE&WT samples. Strikingly, we did not find any up-regulated miRNAs in OsRpp30-KO samples but identified six down-regulated miRNAs. Similarly, between OsRpp30-OE and KO&WT samples, we identified ten down-regulated miRNAs, while no miRNAs were up-regulated. Finally, we identified a total of 41 DEmiRNAs that may play important roles in OsRpp30-mediated disease resistance in rice.

### lncRNAs regulate disease resistance-related genes in ***trans***

To shed light on the potential regulation of lncRNAs on protein-coding genes, we investigated both *trans* and *cis* targets of DElncRNAs. lncRNAs can modulate the expression of distant genes through *trans* mechanisms, involving chromatin remodeling or post-transcriptional regulation [10]. For *trans* targets, we calculated Pearson’s correlation coefficients between DElncRNAs and DEmRNAs, using a similar approach as described in Zhang et al. [27]. Given that lncRNAs can regulate their *trans* targets through various mechanisms, including direct lncRNA-mRNA binding through sequence complementarity, we utilized LncTar [60] to predict putative sequencing binding events between DElncRNAs and their *trans* targets. In total, we identified ten DElncRNA-DEmRNA co-expression pairs between OsRpp30-KO and WT samples, three pairs between OsRpp30-OE and WT samples, and ten pairs between OsRpp30-OE and OsRpp30-KO samples. Given that the OsRpp30-OE and OsRpp30-KO rice lines exhibited the most distinct phenotypes in terms of resistance and susceptibility, our primary focus was on the *trans*-targeting pairs between these two rice lines.

**Fig. 6:**
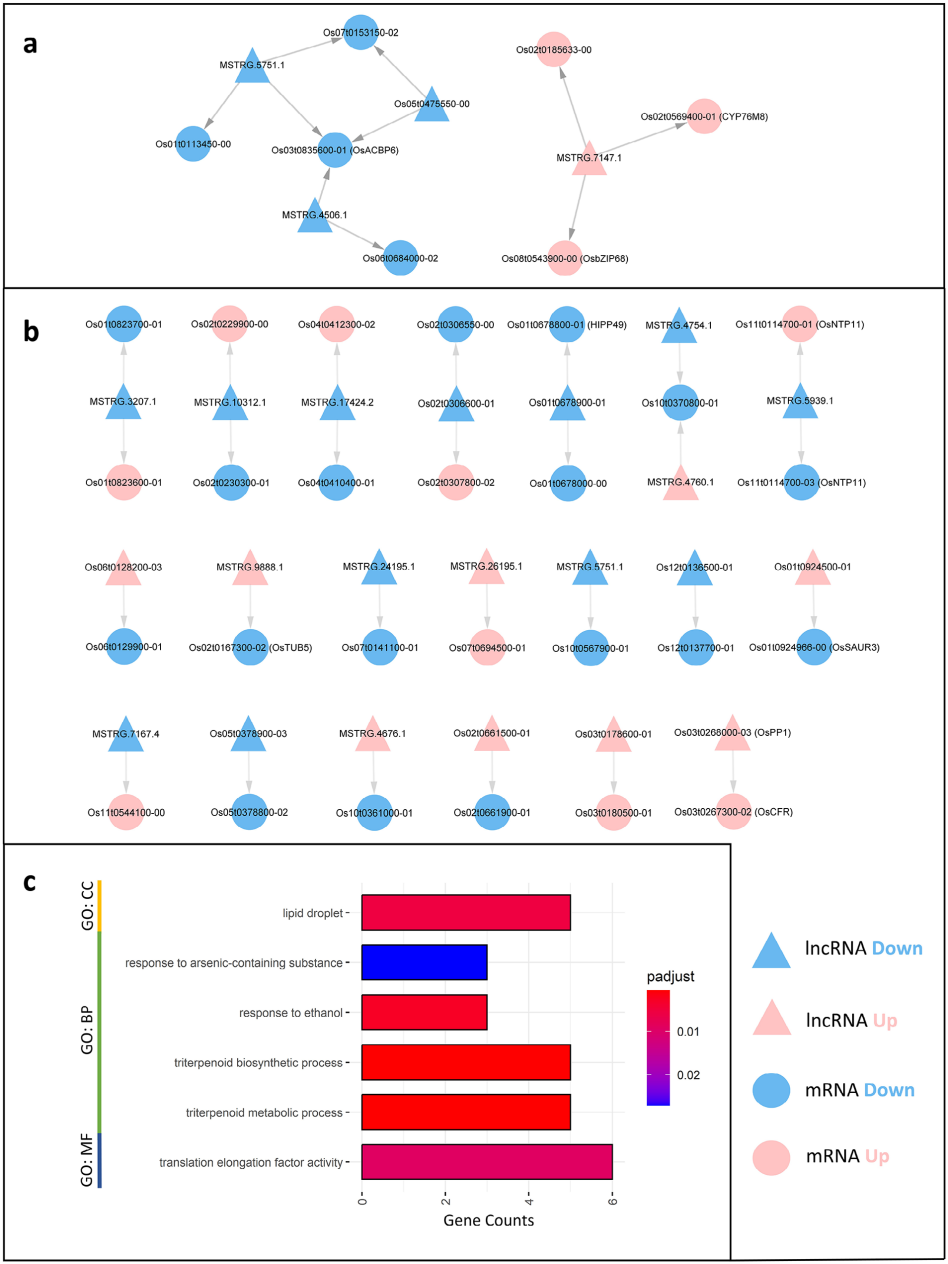
Analysis of DElncRNA *trans* and *cis* targets. **a** The co-expression network of DElncRNAs and their *trans* DEmRNA targets between OsRpp30-OE and OsRpp30-KO. **b** The network of DElncRNAs and their *cis* DEmRNA targets between OsRpp30-OE and OsRpp30-KO. **c** The GO terms enriched for all DElncRNA targets

To further explore the relationship between DElncRNAs and their *trans* targets, we constructed a co-expression network. For instance, we found that the lincRNA MSTRG.5751.1 and its three mRNA targets, *Os01t0113450-00*, *Os07t0153150-02*, and *Os03t0835600-01* were down-regulated in OsRpp30-OE samples (Fig. 6a and Supplementary Table S3, Additional File 1). LncTar analysis confirmed the direct RNA-RNA interaction between MSTRG.5751.1 and *Os01t0113450-00* (See Supplementary Figure S3a, Additional File 1), as well as MSTRG.5751.1 and *Os03t0835600-01* (See Supplementary Figure S3b, Additional File 1), suggesting potential regulation through sequence complementarity. It is worth noting that *Os03t0835600-01* is a transcript of the gene *OsACBP6*, which encodes the Acyl-CoA-binding protein 6. *OsACBP6* is involved in various biological processes, including peroxisomal beta-oxidation and lipid metabolism. Interestingly, a previous study has indicated that *OsACBP6* mutants exhibit up-regulated levels of JA, a product of beta-oxidation in peroxisomes, and JA is involved in regulating plant responses to abiotic and biotic stresses [61]. In our analysis, we found that MSTRG.5751.1, MSTRG.4506.1, and *Os05t0475550-00* act as *trans*-regulators of *OsACBP6* (See Supplementary Figure S3c, Additional File 1), suggesting the potential involvement of *OsACBP6* as an essential lncRNA *trans* target in OsRpp30-mediated disease resistance in rice.

The sense lncRNA MSTRG.7147.1 exhibited up-regulation in OsRpp30-OE samples, along with its three *trans* mRNA targets, *Os02t0185633-00*, *Os08t0543900-00*, and *Os02t0569400-01*. Notably, all three *trans* targets were predicted to have direct RNA-RNA interactions with MSTRG.7147.1 (See Supplementary Figures S3d, S3e and S3f, Additional File 1). It is worth noting that MSTRG.7147.1 and its *trans* targets were exclusively expressed in OsRpp30-OE samples, with no detectable expression in WT or OsRpp30-KO samples. Specifically, *Os08t0543900-00* is a transcript of the gene *OsbZIP68*, a member of the basic leucine zipper (bZIP) transcription factor family. *bZIP68* is highly conserved among plants and plays a role in various stress response-associated biological processes, including cold and oxidative stress tolerance [62, 63]. On the other hand, *Os02t0569400-01* corresponds to a transcript of the gene *CYP76M8*, which encodes a cytochrome P450 (CYP) family protein. *CYP76M8* is involved in the biosynthesis of oryzalexin, an antimicrobial compound produced by rice in response to pathogen attacks [64]. To further investigate the interaction between DElncRNAs and their *trans* targets, we performed sequence alignments and identified a conserved ‘GCG’ tandem repeat motif shared between MSTRG.7147.1 and the *trans* targets *Os02t0185633-00* and *Os08t0543900-00*, suggesting a potential interaction with the same motif binding protein (See Supplementary Figures S4a and S4b, Additional File 1).

### lncRNAs regulate disease resistance-related genes in ***cis***

In addition to their role in regulating *trans* targets, lncRNAs also exert influence on nearby genes, referred to as *cis* targets, by recruiting regulatory proteins to their promoters or by forming R-loops through direct DNA-RNA binding [10]. To identify *cis* targets of DElncRNAs, we adopted a similar approach previously described [15, 27]. By extracting protein-coding genes located within a 100 kb region up and downstream of DElncRNAs, we identified a total of 919 *cis* targets for all DElncRNAs, with 42 exhibiting differential expression in at least two different rice lines. Specifically, we identified 27 DElncRNA-DEmRNA *cis-*targeting pairs between OsRpp30-OE and OsRpp30-KO samples (Fig. 6b and Supplementary Table S4, Additional File 1).

Among the *cis* targets of the DElncRNAs, we identified several genes associated with plant immunity, including *HIPP49*, *Os10t0370800-01*, *Os06t0129900-01*, and *Os10t0361000-01*. Of particular interest, the reference lncRNA *Os01t0678900-01* was found to *cis* targeting the heavy metal-associated isoprenylated protein 49 (*HIPP49*). HIPPs are known to participate in plant responses to heavy metal detoxification and abiotic stress, but certain HIPPs can act as susceptibility factors, negatively impacting disease resistance against pathogens [65]. In OsRpp30-OE samples, both *Os01t0678900-01* and its *cis* target *HIPP49* were downregulated, suggesting a potential mechanism of OsRpp30-mediated disease resistance in rice involving the negative regulation of *HIPP49* by *Os01t0678900-01*. Furthermore, our analysis revealed that *Os01t0678900-01* is located upstream (~ 3 kb) of the *HIPP49* gene, indicating a plausible gene regulatory role of *Os01t0678900-01* on the *HIPP49* promoter region (See Supplementary Figure S5a, Additional File 1).

In addition, we observed that both MSTRG.4760.1 and MSTRG.4754.1 targeted *Os10t0370800-01*, a transcript derived from the gene *Os10g0370800*. This gene encodes a protein that shares similarities with an exo-1,3-beta-glucanase precursor, known for its ability to degrade the cell walls of various pathogens, including bacteria, viruses, and particularly fungi, thereby playing a crucial role in plant resistance against pathogens [66]. Interestingly, we observed a similarity of 26-nt fragment sequence between MSTRG.4754.1 and the second intron of *Os10g0370800* (See Supplementary Figure S4d, Additional File 1). Moreover, the reference lncRNA *Os06t0128200-03* demonstrated specific targeting of the mRNA *Os06t0129900-01*, which encodes a protein resembling a CYP family protein. CYPs are known to play vital roles in hormone signaling pathways and regulate plant response to both biotic and abiotic stresses [67].

Another interesting finding was the targeting of *Os10t0361000-01*, a lipoxygenase gene, by MSTRG.4676.1. Lipoxygenases are enzymes that catalyze polyunsaturated fatty acids into hydroperoxides, ultimately leading to the production of stress response-associated molecules such as JA [68]. A study in maize has demonstrated that the disruption of 9-lipoxygenase results in enhanced resistance against fungal pathogens [69]. Interestingly, a sequence alignment between MSTRG.4676.1 and *Os10t0361000-01* revealed a shared short sequence fragment, ‘GCCGCCGGCCACGA,’ suggesting the presence of a potential binding site for the regulatory proteins (See Supplementary Figure S4e, Additional File 1).

In addition to *Os01t0678900-01*, we identified several DElncRNAs that are located in the promoter regions of their respective *cis* targets, including MSTRG.10312.1 and MSTRG.5751.1. Interestingly, MSTRG.10312.1 not only occupies the promoter region of its *cis* target *Os02t0230300-01* but also functions as a sense lncRNA that partially overlaps with it (See Supplementary Figure S5b, Additional File 1). Conversely, MSTRG.5751.1, classified as a lincRNA, is positioned within the promoter region of *Os10t0567900-01* (See Supplementary Figure S5c, Additional File 1).

To investigate the functions of DElncRNA targets, we performed a GO and KEGG pathway enrichment analysis for both *trans* and *cis* targets. Interestingly, we did not find any significantly enriched GO or KEGG terms among the targets of the down-regulated lncRNAs in the OsRpp30-OE samples. In contrast, we observed significant enrichment of several GO terms among the targets of up-regulated lncRNAs in the OsRpp30-OE samples (Fig. 6c). Notably, one of the enriched GO terms was lipid droplet, representing specialized organelles for lipid storage, primarily in the form of triacylglycerols. Proteins associated with lipid droplets, such as caleosins and steroleosins are involved in plant responses to abiotic and biotic stresses [70]. Additionally, four GO biological processes were enriched, including response to arsenic-containing substance, response to ethanol, triterpenoid biosynthetic, and triterpenoid metabolic processes. Triterpenoids are natural plant products that contribute to plant defense against pathogens and herbivores [71]. Finally, in terms of GO molecular function, we found enrichment in a single term: translation elongation factor activity.

### lncRNAs regulate Disease resistance-related genes through a lncRNA-miRNA-mRNA competing endogenous RNA (ceRNA) network

**Fig. 7:**
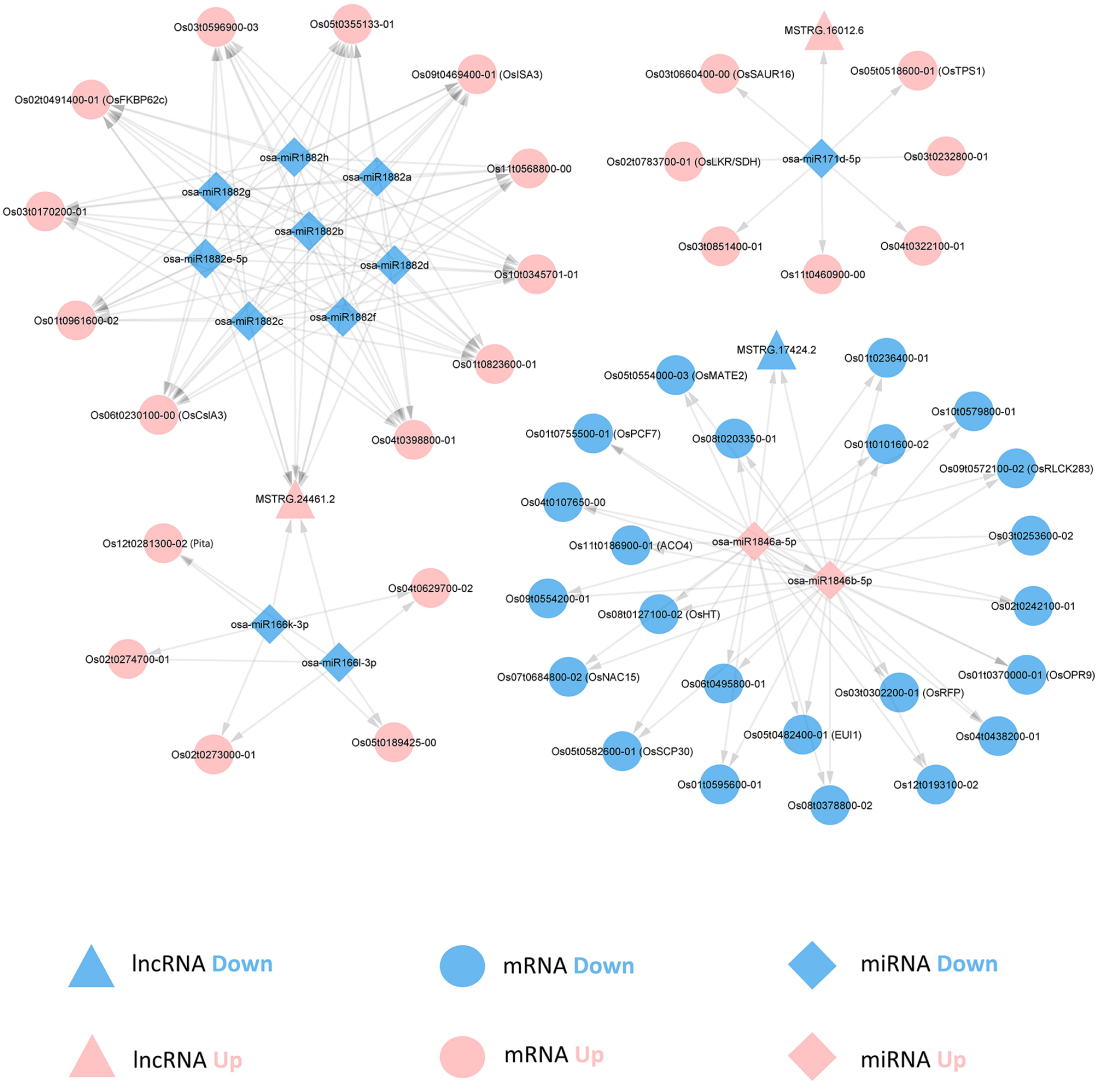
lncRNA-miRNA-mRNA competing endogenous RNA (ceRNA) network between OsRpp30-OE and OsRpp30-KO. RNA transcripts that were up- and down-regulated in OsRpp30-OE samples are in red and blue colors, respectively. lncRNA, miRNA, and mRNA are represented by triangle, diamond, and ellipse, respectively

In plants, miRNAs can target both lncRNAs and mRNAs, reducing their expression through RNA transcript cleavage. Additionally, lncRNAs can act as sponges, competing with mRNAs for MREs, thereby sequestering miRNAs and indirectly modulating the expression of mRNAs. To gain deeper insights into how lncRNAs orchestrate the regulation of mRNAs through this ceRNA mechanism, we constructed a ceRNA network that integrates the DEmiRNAs and their respective differentially expressed targets from the OsRpp30-OE and OsRpp30-KO samples (Fig. 7 and Supplementary Table S5, Additional File 1). The representative predicted DEmiRNA-DElncRNA and DEmiRNA-DEmRNA targeting pairs were shown in Supplementary Tables S6 and S7 of Additional File 1, respectively.

Among the up-regulated lncRNAs in OsRpp30-OE samples, MSTRG.24461.2 emerged as a target for several down-regulated miRNAs, including members of the *osa-miR166* family (*osa-miR166k-3p* and *osa-miR166l-3p*) and *osa-miR1882* family (*osa-miR1882a*, *osa-miR1882b*, *osa-miR1882c*, *osa-miR1882d*, *osa-miR1882e-5p*, *osa-miR1882f*, *osa-miR1882g*, and *osa-miR1882h*). Previous studies have demonstrated that *osa-miR166* is involved in rice defense against fungi and viruses [58, 59]. In addition to MSTRG.24461.2, *osa-miR166* and *osa-miR1882* also targeted several OsRpp30-OE up-regulated mRNAs within the ceRNA network, such as *Pita*, *Os03t0170200-01*, and *OsCSLA3*. Both *Pita* and MSTRG.24461.2 were up-regulated in the OsRpp30-OE samples, competing for the limited number of *osa-miR166* transcripts. On the contrary, *Os03t0170200-01* and *OsCSLA3* competed with MSTRG.24461.2 for *osa-miR1882*. *Os03t0170200-01* is a transcript of the gene *Os03g0170200*, which shares similarities with the MADS-box transcription factor 30. MADS-box transcription factors are involved in different development processes and stress responses in plants [72]. For instance, the knockdown of *OsMADS26* in rice plants enhances resistance against rice blast and bacterial blight pathogens [73]. *OsCSLA3* is a member of cellulose synthases, which are crucial proteins involved in cell wall formation and plant disease resistance. Disruption of cellulose synthases A3 in *A. thaliana* has been shown to enhance pathogen resistance by activating JA and ET signaling pathways [74]. Furthermore, inhibiting cellulose synthases necessary for secondary cell wall integrity increases pathogen resistance in *A. thaliana* through the abscisic acid (ABA) pathway [75].

MSTRG.16012.6, an up-regulated lncRNA in the OsRpp30-OE samples, was found to be targeted by the down-regulated miRNA, *osa-miR171d-5p*. In the ceRNA network, several up-regulated mRNAs competed with MSTRG.16012.6 for *osa-miR171d-5p*. For instance, *Os02t0783700-01* is a transcript of the bifunctional gene lysine ketoglutarate reductase/saccharopine dehydrogenase (OsLKR/SDH). LKR/SDH serves as a key enzyme in the saccharopine pathway for lysine catabolism, which is induced in response to abiotic or biotic stresses [76, 77]. Another example is *OsTPS1*, which encodes a trehalose 6-phosphate synthase responsible for the biosynthesis of trehalose. Trehalose plays a crucial role in abiotic stress response in plants, and studies have shown that overexpression of *OsTPS1* increases trehalose concentration and enhances rice tolerance to cold, salt, and drought stress [78]. In addition, trehalose contributes to resistance against bacterial wilt disease in tomatoes [79].

MSTRG.17424.2 was identified as a target of the up-regulated *osa-miR1846* family miRNAs, *osa-miR1846a-5p* and *osa-miR1846b-5p*. Our study revealed that several down-regulated mRNAs competed with MSTRG.17424.2 for *osa-miR1846*, many of which were associated with plant immunity. One of the targets, *OsMATE2*, encodes a multi-antimicrobial extrusion protein known to negatively regulate plant immunity in response to bacterial pathogens in *A. thaliana* [80]. The down-regulation of *OsMATE2* in the OsRpp30-OE samples suggests its potential role in OsRpp30-mediated disease resistance. Another target, *Os04t0107650-00* is a transcript of the gene *Os04g0107650*, which shares similarities with arginine decarboxylase (ADC). ADC plays a vital role in plant defense against bacterial pathogens, as evidenced by the up-regulation of salicylic acid (SA)- and JA-dependent pathogenesis-related genes in *A. thaliana* with down-regulated *ADC2* [81]. *OsNAC15* is a NAC (NAM, ATAF1/2, and CUC2) domain-containing protein-coding gene, belonging to a large transcription factor family that plays critical roles in plant stress response, including biotic infections and abiotic stresses [82]. Previous studies have demonstrated that overexpression of the NAC family gene *OsNAC6* enhances rice resistance against blast disease [83]. Although the function of *OsNAC15* in biotic stress response is currently not well understood, a recent study showed that *OsNAC15* contributes to abiotic stress response in rice, such as zinc deficiency and cadmium stress [84]. *EUI1* (elongated uppermost internode 1) is a Gibberellin (GA) deactivating enzyme and it is involved in rice defense against bacterial blight [85]. Notably, *miR1846* negatively regulates ACO proteins, which regulate the biosynthesis of ET, thereby affecting the plant defense-associated ET signaling pathway [49]. The consistent down-regulation of *ACO4* by *osa-miR1846* suggests a putative regulatory network involving ET in OsRpp30-mediated disease resistance in rice.

## Discussion

RNase P is a conserved ribonucleoprotein complex found in all three life kingdoms, with its primary function being the cleavage of the 5’ end of precursor tRNAs and the maturation of tRNAs [29–31]. It is composed of an RNA molecule and several protein subunits, ranging from one in bacteria to nine to ten in eukaryotes [86]. However, except for a few exceptional cases, such as human mitochondrial RNase P, only the RNA component is required for catalytic activity, while the protein subunits primarily serve auxiliary roles such as tRNA recognition and proper folding of the catalytic RNA [31, 87]. In addition to their role in precursor tRNA cleavage, RNase P protein subunits are involved in various biological processes, including chromatin assembly, transcriptional regulation, and DNA damage repair [31, 88, 89]. For instance, human Rpp29 and Rpp21 proteins are implicated in DNA double-strand break repair, whereas other RNase P subunits, such as Rpp14, Rpp25, and Rpp38, do not respond to DNA breaks [89]. These findings suggest that the protein subunits of RNase P may have functions beyond tRNA maturation. Indeed, as a conserved RNase P protein subunit, Rpp30 has been found to play essential roles, and dysregulation of Rpp30 has been linked to diseases, such as female sterility in *Drosophila* and cancers in humans [90–92]. Our previous study also indicated that the rice OsRpp30 positively regulates plant immunity, and the histone deacetylase HDT701 may serve as an upstream regulator of OsRpp30 [32]. In addition to the involvement in tRNA processing, mounting evidence demonstrates that RNase P is also implicated in processing small RNAs and lncRNAs [93–95]. For instance, the lncRNA MALAT1 (Metastasis-Associated Lung Adenocarcinoma Transcript 1) can be recognized and processed by RNase P at its 3’ end [96]. Additionally, the antisense lncRNA HRA (Hiding in Reading-Frame Antisense) was identified as a substrate of RNase P in *Saccharomyces cerevisiae* [97]. These non-canonical functions of RNase P inspire us to consider the possibility that there may be lncRNAs and miRNAs downstream of the OsRpp30-mediated disease resistance pathway that regulates the expression of defense-related genes in rice.

This current study sheds light on the role of lncRNAs and miRNAs in OsRpp30-mediated disease resistance in rice through the regulation of defense-associated mRNAs. Despite not encoding proteins, lncRNAs function as essential regulatory molecules. They are known to modulate gene expression through histone modification and transcriptional/post-transcriptional interference [10]. In plants, lncRNAs participate in diverse biological processes, including flowering, photoperiod-sensitive male sterility, and immunity [12–15, 26]. As crucial regulators of gene expression, lncRNAs can regulate neighboring genes in *cis* or distant genes in *trans* [20–23]. Moreover, lncRNAs serve as decoys for miRNAs through sequence complementarity, thereby mitigating their impact on mRNA targeting [26]. In this study, we leveraged the annotated lncRNAs in the Rice Annotation Project Database (RAP-DB) [98] and identified 948 novel lncRNAs with high confidence in rice samples using our new tool LncDC [33], significantly expanding the repertoire of known lncRNAs in rice plants. Certain miRNAs, such as *miR169* and *miR398b*, have been implicated in rice disease resistance [6, 7]. Furthermore, studies have reported differential expression of lncRNAs in response to pathogen infection in rice plants [27, 28]. However, there remains limited research on the interplay among lncRNAs, miRNAs, and mRNAs during disease resistance in rice. To the best of our knowledge, this study is the first to profile the expression of lncRNAs, miRNAs, and mRNAs in a collection of defense mutants in rice plants, contributing to a better understanding of the interactions among different categories of RNAs involved in OsRpp30-mediated disease resistance.

miRNAs have been implicated in various biological processes, including their role in modulating rice immunity by targeting mRNAs of defense genes [5]. Several of the DEmiRNAs we identified were defense-associated miRNAs, such as *osa-miR169*, *osa-miR398b*, and *osa-miR528*. For instance, *osa-miR169* negatively regulates rice defense against blast disease by suppressing the expression of *NF-YA* genes [6, 7]. The up-regulation of *osa-miR169i-5p.2* in the OsRpp30-KO samples suggests that *OsRpp30* may regulate rice immunity by inhibiting *osa-miR169*. On the contrary, *osa-miR398b* acts as a positive regulator in rice blast resistance by enhancing H_2_O_2_ production, and we observed its down-regulation in OsRpp30-KO samples [7]. Notably, *miR398b* exhibits opposing roles in plant defense against fungi and bacteria [5]. It promotes rice defenses against fungi but negatively affects *A. thaliana* immunity against bacteria. This dual function in regulating immunity regulatory mechanisms may arise from long-term competition and coevolution between plants and pathogens. Additionally, *osa-miR528* serves as a negative regulator in rice resistance against viruses [51, 52]. However, we observed the up-regulation of osa-miR528-5p in the OsRpp30-OE resistant rice lines, indicating its positive regulatory function in rice immunity against fungal and bacterial pathogens. Therefore, *osa-miR528* may also exhibit dual roles in plant immunity, depending on the specific pathogens involved.

Plants employ two distinct strategies for pathogen recognition: pathogen-associated molecular pattern (PAMP)-triggered immunity (PTI) and effector-triggered immunity (ETI). Activation of PTI and ETI initially triggers distinct amplitudes and dynamics of defense gene expression but eventually converges into many similar downstream responses, including the production of reactive oxygen species, activation of kinase signaling, up-regulation of hormone-associated genes, synthesis of antimicrobial compounds, and reprogramming of cell wall components [4, 28]. lncRNAs possess the ability to regulate target genes through *cis* or *trans* targeting, acting as decoys for miRNAs and indirectly influencing target gene expression. In our study, we observed that lncRNAs can modulate rice immunity at different stages by directly or indirectly regulating defense-associated genes. Notably, NBS-LRR proteins serve as key receptors for triggering ETI, either through direct interaction with pathogen effectors or indirect association. Differential expression of several NBS-LRR genes, including *Pita*, *Os06g0707350*, and *Os08g0543500*, was observed between OsRpp30-OE and OsRpp30-KO samples. *Pita*, a disease resistance gene against rice blast disease, was found to be up-regulated in OsRpp30-OE samples [38, 39]. Our findings suggest that *Pita* is involved in the lncRNA-miRNA-mRNA ceRNA network and is regulated by both the miRNA *osa-miR166* and the lncRNA MSTRG.24461.2, further indicating the involvement of lncRNAs in OsRpp30-mediated rice immunity and activation of ETI.

After pathogen recognition, plant hormone pathways, including SA, JA, ABA, and ET, are activated in response to pathogen attack. However, utilizing metabolites for disease resistance incurs a fitness cost on plants, negatively impacting development and seed production [99]. The intricate hormone networks govern plant immunity by striking a balance between development and defense [5]. In our study, we identified lncRNA targets involved in regulating hormone signaling pathways. For instance, *OsACBP6*, an Acyl-CoA-binding protein associated with peroxisomal beta-oxidation, acts as a negative regulator of JA expression [61]. Our findings demonstrate the down-regulation of *OsACBP6* in OsRpp30-OE samples and reveal its targeting by lncRNAs MSTRG.5751.1, MSTRG.4506.1, and *Os05t0475550-00*.

lncRNAs play a role in regulating hormone signaling pathways through interactions among lncRNAs, miRNAs, and mRNAs. One example is *Os04g0107650*, a member of the ADC protein family, which has been previously demonstrated to exert negative regulation on the SA and JA pathways [81]. *EUI1*, an inhibitor of the GA pathway, contributes to rice immunity against bacterial blight [85]. Additionally, *ACO4*, an ACO protein, is involved in the biosynthesis of ET and contributes to the activation of the ET pathway [49]. Our findings reveal the down-regulation of *Os04g0107650*, *EUI1*, and *ACO4* in OsRpp30-OE samples, and they serve as targets of both the miRNA *osa-miR1846* and the lncRNA MSTRG.17424.2 through the ceRNA network. These results suggest that lncRNAs mediate hormone signaling pathways associated with rice immunity through *cis*, *trans*, and ceRNA network targeting mechanisms.

In addition, we observed that lncRNAs regulate certain defensive proteins with antimicrobial properties. One such protein is *Os10g0370800*, which shares similarities with a precursor involved in the biosynthesis of beta-1,3-glucanases. These glucanases are important pathogenesis-related proteins that play a crucial role in attacking pathogens, particularly fungi, by degrading their cell walls [66]. Interestingly, *Os10g0370800* exhibited down-regulation in the OsRpp30-OE samples and was identified as a *cis* target of two lncRNAs, namely MSTRG.4760.1 and MSTRG.4754.1. Another protein of interest is *OsTPS1*, a trehalose synthase that regulates the biosynthesis of trehalose. Trehalose is known to contribute to plants’ stress response and disease resistance [78, 79]. In our study, we observed the up-regulation of *OsTPS1* in the OsRpp30-OE samples, and its regulation involved both the miRNA *osa-miR171d-5p* and the lncRNA MSTRG.16012.6. These findings suggest that lncRNAs may enhance rice immunity by promoting the expression of antimicrobial compounds.

Transcription factors play a critical role in regulating gene transcription, particularly in response to pathogen perception [100]. In our study, we investigated the regulatory role of lncRNAs on transcription factors. *OsbZIP68*, a bZIP transcription factor implicated in plant stress response, showed up-regulation in OsRpp30-OE samples and was targeted by the *trans*-acting lncRNA MSTRG.7147.1 [62, 63]. Another noteworthy transcription factor is *OsNAC15*, a member of the NAC transcription factor family, which plays a pivotal role in plant immunity. Numerous NAC transcription factors have been shown to either enhance or inhibit plant immune responses by regulating pathogenesis-related genes and hormone pathways [82, 83, 100]. In our study, we observed down-regulation of *OsNAC15* in OsRpp30-OE samples, and its expression was regulated by both the miRNA *osa-miR1846* and the lncRNA MSTRG.17424.2. Collectively, our findings indicate that lncRNAs can directly modulate the activity of transcription factors through *trans*-regulation or indirectly influence their expression by acting as miRNA decoys.

lncRNAs can regulate gene transcription by facilitating transcription factor recruitment and histone modification [10]. In our study, we observed several DElncRNAs located in proximity to the promoter regions of their *cis* targets. For instance, the lncRNA *Os01t0678900-01* was found in the vicinity of the *HIPP49* promoter region and positively regulated its expression through a *cis*-acting mechanism. HIPPs are key proteins involved in abiotic stress responses, including heavy metal stress, and they also negatively regulate disease resistance in plants [65]. The down-regulation of both *Os01t0678900-01* and *HIPP49* in the OsRpp30-OE samples implies that lncRNAs might modulate plant immunity by exerting regulatory effects at the promoter region of their target genes. A well-known example demonstrating lncRNAs regulating their targets through promoter interactions is the lncRNA *HOTTIP*, which recruits histone methyltransferase complexes to the promoter regions of its target genes, consequently leading to H3K4me3 modification and activation of transcription [20]. In addition to *Os01t0678900-01*, we also identified DElncRNAs MSTRG.10312.1 and MSTRG.5751.1 positioned in proximity to the promoter regions of their respective targets despite their targets not being directly associated with defense-related genes.

*OsRpp30* has been previously reported to interact with the histone deacetylase *HDT701* in rice, indicating its involvement in the epigenetic regulation of rice immunity [32, 42]. While several histone modification-associated proteins, such as *HDT701*, *OsSET30*, and *SDG714*, exhibited differential expression across the different rice lines, none were identified as targets of DElncRNAs. However, our analysis revealed that *OsSET30* and *SDG714* were targeted by miRNAs, specifically *osa-miR166e-5p* and *osa-miR531b*, respectively. Both *OsSET30* and *SDG714* are methyltransferases, with *SDG714* specifically functioning as a histone H3K9 methyltransferase associated with transcription suppression [101]. Interestingly, *OsSET30* was down-regulated in OsRpp30-KO samples, while its targeting miRNA, *osa-miR166e-5p*, was up-regulated in the same samples. Conversely, *SDG714* was up-regulated in OsRpp30-KO samples, despite the down-regulation of its targeting miRNA, *osa-miR531b*. These observations suggest a potential regulatory role of miRNAs in histone modification during OsRpp30-mediated disease resistance.

Overall, our analysis provides comprehensive profiling of lncRNAs, miRNAs, and mRNAs in OsRpp30-OE, OsRpp30-KO, and WT samples, shedding light on the intricate interactions among these three RNA groups in the context of disease resistance. While it is important to note that the identified lncRNAs have not been validated using RT-PCR (reverse transcription-polymerase chain reaction) or other wet-lab approaches previously described [102], we are highly confident in the lncRNAs we identified based on the following reasons: (1) The mapping rates of sequencing reads averaged approximately 94%, indicating the high quality of our sequencing libraries. (2) We employed stringent criteria in the selection of lncRNA candidates. For instance, to minimize the false positive rate associated with single exon transcripts assembled by StringTie, we exclusively retained transcripts with two or more exons as lncRNA candidates [103]. (3) Many of the identified lncRNAs exhibited high similarity to lncRNAs present in existing databases, and some of the DElncRNAs we discovered were previously reported as disease-responsive lncRNAs in rice [15, 104]. Despite the absence of wet-lab validation, these factors collectively enhance the reliability and validity of our identified lncRNAs.

## Conclusions

This study represents a comprehensive investigation of the expression profiles of lncRNAs, miRNAs, and mRNAs in a collection of defense mutants in rice, shedding new light on their roles in OsRpp30-mediated disease resistance. The results underscore the significance of lncRNAs as crucial regulators in this context, as evidenced by their differential expression patterns across OsRpp30-OE, OsRpp30-KO, and WT rice samples. These DElncRNAs exert regulatory control over DEmRNAs that participate in pathogen recognition, hormone pathways, and other downstream responses in rice immunity by targeting them through *cis*, *trans*, or ceRNA networks. We anticipate that these findings will enhance our understanding of the functions of OsRpp30 and provide candidate lncRNA-miRNA-mRNA interaction pairs for future research aiming to validate the functional roles of these lncRNAs.

## Methods

### Plant materials and growth conditions

The generation of the OsRpp30-OE and OsRpp30-KO transgenic lines was described previously [32]. The seeds of two transgenic lines and the WT Nipponbare were sterilized by treatment with 75% (v/v) ethanol for 1 min, followed by immersion in 2% (w/v) NaCl for 40 min. The treated seeds were washed with sterile water and germinated on 1/2 Murashige and Skoog (MS) medium for 8–9 days at 26 °C with a 12-h/12-h light/dark photoperiod. Subsequently, the seedlings were transferred to soil and grown in a growth chamber at 26 °C and 80% relative humidity with a 12-h/12-h light/dark photoperiod. The leaves of 4-week-old WT, OsRpp30-OE, and OsRpp30-KO plants were collected, flash-frozen in liquid nitrogen, and stored at − 80 °C. Two biological replicates were obtained for each sample.

### RNA library construction and sequencing

For mRNAs and lncRNAs, total RNA (1.5 µg per sample) was extracted from plants in different groups. To remove rRNAs, the Ribo-Zero rRNA Removal Kit (Epicentre, Madison, WI, USA) was used following the manufacturer’s instructions. Subsequently, six sequencing libraries were prepared using the NEBNext Ultra Directional RNA Library Prep Kit for Illumina (NEB, USA). The resulting libraries were sequenced on the Illumina Hiseq 2500 platform with paired-end reads generated.

For small RNAs, a total amount of 1.5 µg RNA per sample was used for library construction. The NEBNext Ultra Small RNA Sample Library Prep Kit for Illumina (NEB, USA) was employed according to the manufacturer’s guidelines to generate sequencing libraries. The quality of the constructed libraries was assessed using the Agilent Bioanalyzer 2100 system. Sequencing was performed on the Illumina Hiseq X Ten platform with single-end reads generated.

### Identification of novel lncRNAs

The raw RNA-Seq reads from WT, OsRpp30-OE, and OsRpp30-KO rice samples (two biological replicates for each sample) were subject to quality control using FastQC [105], followed by preprocessing steps that involved the removal of adapters, low-quality ends, and reads containing poly-N bases. The clean reads were then mapped to the rice reference genome IRGSP-1.0 [106] using HISAT2 v2.2.0 [107]. Transcript assembly was performed using StringTie v2.1.2 [108]. To ensure that the assembled transcripts were expressed in our rice samples, we removed transcripts with FPKM values less than 0.1. Additionally, to minimize false positives produced by StringTie for single-exon transcripts, we retained only transcripts with a minimum of two exons for further analysis [103]. In addition, given that lncRNAs are defined as RNAs longer than 200 nts, we excluded transcripts shorter than 200 nts from the analysis.

The selected transcripts were compared with known rice gene annotations obtained from RAP-DB using Cuffcompare [98, 109]. Transcripts with Cuffcompare generated classification codes ‘i’ (intronic transcript), ‘o’ (transcript overlapped with an exon of a gene), ‘x’ (transcript overlapped with an exon of a gene on the opposite strand), and ‘u’ (intergenic transcript) were selected as lncRNA candidates. These lncRNA candidates were then subject to prediction using both LncDC v1.3.3 and Pfam v35.0 [33, 34]. Only candidates predicted as lncRNAs by both programs were considered confident lncRNAs. BLAST v2.9.0 [110] was used to compare the lncRNAs against existing lncRNA databases, including NONCODE v6, CANTATAdb v2, and RiceLncPedia [35–37]. To maintain consistency in naming, the predicted lncRNAs were assigned the prefix ‘MSTRG’. Additionally, prefixes, such as ‘Os’, ‘NONOSA’, ‘CNT’, ‘Osa’, and ‘TCONS’ were used for lncRNAs from RAP-DB, NONCODE, CANTATAdb, RiceLncPedia, and the study by Yu et al. [15], respectively.

In addition to the predicted novel lncRNAs, we extracted 2,063 annotated reference lncRNAs obtained from RAP-DB [98] (biotype = nontranslating_CDS, length > = 200 nts) for further analyses.

### Identification of miRNAs

The raw small RNA-Seq reads were subject to quality control using FastQC [105], followed by removing adapters, low-quality ends, reads containing poly-N bases, and reads shorter than 18 nts or greater than 30 nts. Subsequently, Bowtie v1.3.1 [111] was used to map the clean reads to the same rice reference genome IRGSP-1.0 [106]. The mapped reads were compared to known mature and precursor miRNAs from miRbase v22.1 [112] by miRDeep2 v0.1.3 [113]. miRDeep2 was also used for the quantification of miRNA expression levels in different samples.

### Differential expression analysis of lncRNAs, mRNAs, and miRNAs

Stringtie v2.1.2 [108] was used to quantify the transcript abundance of lncRNAs and mRNAs in samples from the three rice lines. On the other hand, miRDeep2 v0.1.3 [113] was used to quantify the expression of miRNAs. The read counts data was then subject to DESeq2 [114] to identify DElncRNAs, DEmRNAs, and DEmiRNAs among the WT, OsRpp30-OE, and OsRpp30-KO samples. Differential expressions were determined based on the criteria of |log_2_(Fold Change)| > 1 and p-adjusted value < 0.05. PCA analysis and expression plots were generated using DESeq2. Volcano plots for DElncRNAs, DEmRNAs, and DEmiRNAs were created using ggplot2 [115]. Additionally, FPKM values were calculated for DElncRNAs across different rice lines and heatmaps were generated using pheatmap (https://cran.r-project.org/web/packages/pheatmap/index.html).

### Target prediction of DElncRNAs and construction of lncRNA-mRNA interaction network

We performed predictions for both *trans* and *cis* mRNA targets of the DElncRNAs. The correlation between DElncRNAs and DEmRNAs across all six samples was assessed by calculating Pearson’s correlation coefficients using the R package psych [116]. *Trans*-targeting pairs were selected based on |R| > 0.9 and p-adjust value < 0.05. We employed LncTar to predict RNA-RNA interactions between DElncRNAs and their targeted DEmRNAs, with a criterion of normalized delta G (ndG) < -0.1 [60].

Additionally, we selected adjacent protein-coding genes within a 100 kb region upstream and downstream of DElncRNAs as their *cis* mRNA targets. The resulting interaction network, which encompasses DElncRNAs, their *trans-targeted* DEmRNAs, and *cis-targeted* DEmRNAs was visualized using Cytoscape [117].

### Enrichment analysis

We conducted GO [118] and KEGG [119] pathway enrichment analyses for DEmRNAs and the targets of DElncRNAs using g:Profiler [120]. Three GO categories were considered in the GO enrichment analysis: molecular function, cellular component, and biological process. The GO terms with an adjusted p-value < 0.05 were considered significantly enriched terms.

### Target prediction of DEmiRNAs and construction of lncRNA-miRNA-mRNA ceRNA network

We used psRNATarget and RNAhybrid (< -20 kcl/mol) to predict the interactions between DEmiRNAs and DElncRNAs as well as DEmiRNAs and DEmRNAs [121, 122]. Only the targeting pairs concordantly predicted by both programs were selected for constructing the lncRNA-miRNA-mRNA ceRNA network. The constructed network was visualized using Cytoscape (https://cytoscape.org/).

### Electronic supplementary material

Below is the link to the electronic supplementary material.

Additional file 1: Supplementary figures and tables.

Additional file 2: List of predicted lncRNAs.

Additional file 3: Sequence comparison between predicted lncRNAs and lncRNAs from other databases.

Additional file 4: List of DElncRNAs.

Additional file 5: List of DEmRNAs.

Additional file 6: List of DEmiRNAs.

## Data Availability

The raw RNA-Seq and small RNA-Seq data generated in this study have been deposited in the Sequence Read Archive (SRA) database of the National Center for Biotechnology Information (NCBI) with the BioProject ID PRJNA993287.

## List of Abbreviations

lncRNA: Long non-coding RNA
lincRNA: Long intergenic non-coding RNA
miRNA: MicroRNA
ceRNA: Competitive endogenous RNA
OsRpp30: Oryza sativa RNase P protein 30
WT: Wildtype
OsRpp30-OE: *OsRpp30* overexpression
OsRpp30-KO: *OsRpp30* knockout
DE: Differentially expressed
nt: Nucleotide
*A. thaliana*: *Arabidopsis thaliana*
*Xoo*: *Xanthomonas oryzae pv. Oryzae*
*M. oryzae*: *Magnaporthe oryzae*
JA: Jasmonate
SA: Salicylic acid
ET: Ethylene
GA: Gibberellin
ABA: Abscisic acid
RNA-Seq: RNA sequencing
MRE: miRNA response element
FPKM: Fragments per kilobase of transcript per million mapped fragments
NBS-LRR: Nucleotide-binding site leucine-rich repeat
SET: Su(var)3–9, enhancer of zeste, and trithorax
GO: Gene ontology
KEGG: Kyoto Encyclopedia of Genes and Genomes
NF-YA: Nuclear factor Y-A
ACO: 1-aminocyclopropane-1-carboxylic acid oxidase
ADC: Arginine decarboxylase
CYP: Cytochrome P450
HIPP: Heavy metal-associated isoprenylated protein
ETI: Effector-triggered immunity
PAMP: Pathogen-associated molecular pattern
PTI: PAMP-triggered immunity
