# Supplementary material for: Transcriptome analysis reveals a lncRNA-miRNA-mRNA regulatory network in OsRpp30-mediated disease resistance in rice
Source: BMC Genomics. 2023 Oct 26;24:643. doi: 10.1186/s12864-023-09748-w (PMC10604448; doi:10.1186/s12864-023-09748-w)
Supplement: Supplementary file 1 — Additional file 1: Supplementary figures and tables. [file 12864_2023_9748_MOESM1_ESM.docx]

**Transcriptome analysis reveals a lncRNA-miRNA-mRNA regulatory network in OsRpp30-mediated disease resistance in rice**

Minghua Li^1^, Wei Li^2^, Meixia Zhao^3^, Zhiqiang Li^4^, Guo-Liang Wang*’^2^, Wende Liu*’^4^, Chun Liang*’^1^

1. Department of Biology, Miami University, Oxford, OH, 45056, USA

2. Department of Plant Pathology, Ohio State University, Columbus, OH, 43210, USA

3. Department of Microbiology and Cell Science, University of Florida, Gainesville, FL, 32611, USA

4. State Key Laboratory for Biology of Plant Diseases and Insect Pests, Institute of Plant Protection, Chinese Academy of Agricultural Sciences, Beijing 100193, China

* Co-corresponding authors

**Supplementary Materials**

[Supplementary Figure S1. Principal component analysis (PCA) of the sequencing data.](#_lv82wozcsjzq) 2

[Supplementary Figure S2. Gene ontology (GO) and Kyoto Encyclopedia of Genes and Genomes (KEGG) pathway enrichment analyses.](#_y7idligyhd60) 3

[Supplementary Figure S3. Representative sequence fragments of RNA-RNA interaction between DElncRNAs and their trans or cis targets predicted by LncTar.](#_rh56pps8x3q5) 4

[Supplementary Figure S4. Alignment results between DElncRNAs and their trans or cis targets.](#_xujtny4qo29c) 5

[Supplementary Figure S5. The genomic coordinates of DElncRNAs and their cis targets in integrative genomics viewer (IGV).](#_s2poe8mtho64) 6

[Supplementary Table S1. The summary of the RNA-Seq and small RNA-Seq data.](#_u73dhdz2sk99) 7

[Supplementary Table S2. The predicted novel lncRNAs and their highly similar lncRNAs in NONCODE, CANTATAdb, and RiceLncPedia databases.](#_phjfuauqozyg) 7

[Supplementary Table S3. The summary of the genomic coordinates and expression level of DElncRNA-DEmRNA trans-targeting pairs.](#_lrv03a48wmr9) 9

[Supplementary Table S4. The summary of the genomic coordinates and expression level of DElncRNA-DEmRNA cis-targeting pairs. 1](#_7q0mykuq4del)0

[Supplementary Table S5. The summary of the genomic coordinates and expression level of the RNAs in the lncRNA-miRNA-mRNA competing endogenous RNA (ceRNA) network.. 1](#_o987n0tbmdeb)3

[Supplementary Table S6. The representative DEmiRNA-DElncRNA targeting pairs predicted by psRNATarget. 1](#_ggfnalah64qx)6

[Supplementary Table S7. The representative DEmiRNA-DEmRNA targeting pairs predicted by psRNATarget. 1](#_605fopsaeyim)7


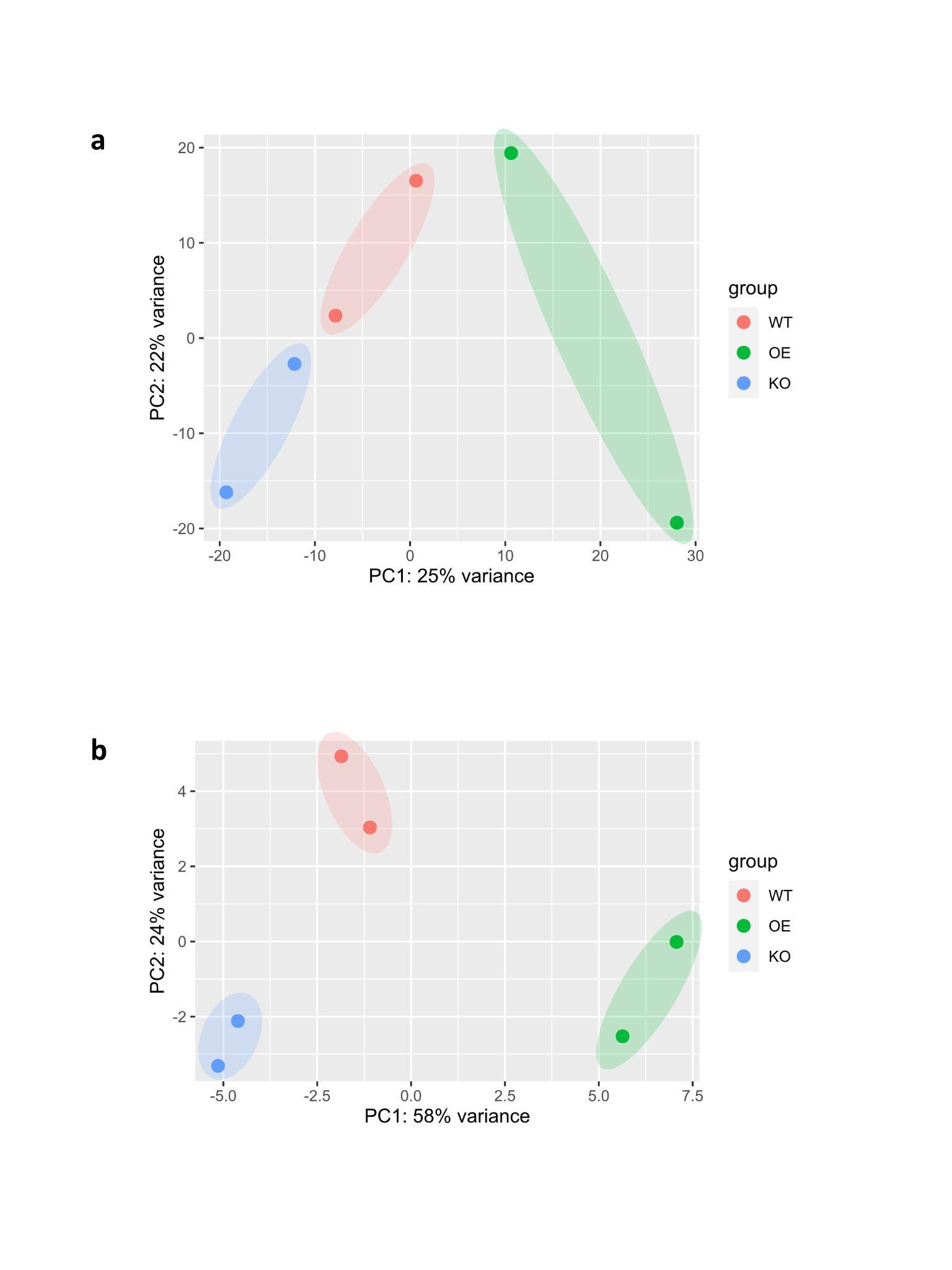


## Supplementary Figure S1. Principal component analysis (PCA) of the sequencing data. **a**. PCA plot for RNA expression profile of lncRNAs and mRNAs in samples from wildtype (WT), OsRpp30 overexpression (OsRpp30-OE), and OsRpp30 knockout (OsRpp30-KO) groups. **b**. PCA plot for RNA expression profile of miRNAs in samples from different experimental groups.


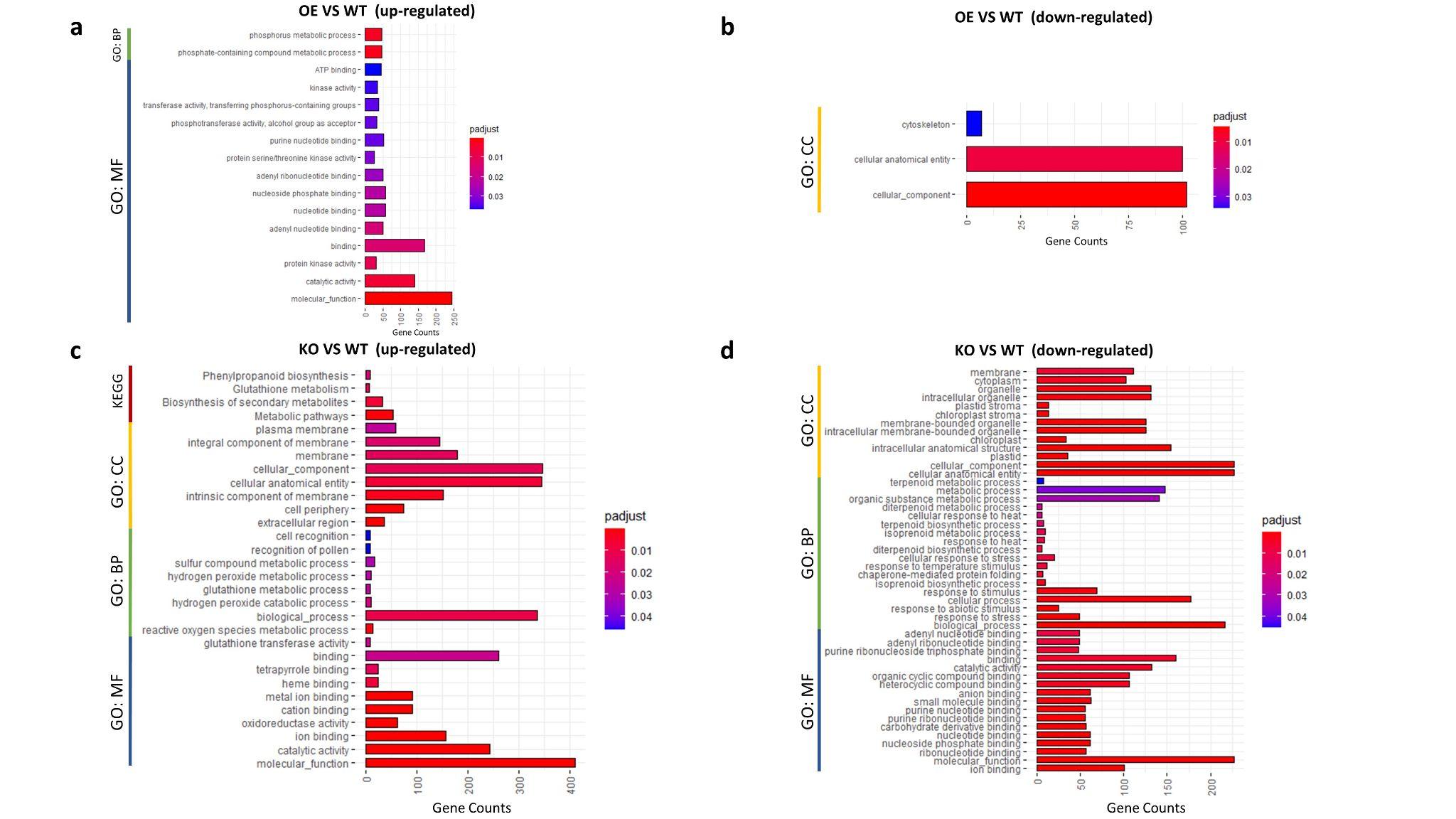


## Supplementary Figure S2. Gene ontology (GO) and Kyoto Encyclopedia of Genes and Genomes (KEGG) pathway enrichment analyses. **a.** The GO terms enriched for up-regulated mRNAs between OsRpp30-OE and WT. **b.** The GO terms enriched for down-regulated mRNAs between OsRpp30-OE and WT. **c.** The GO terms and KEGG pathways enriched for up-regulated mRNAs between OsRpp30-KO and WT. **d.** The GO terms enriched for down-regulated mRNAs between OsRpp30-KO and WT.


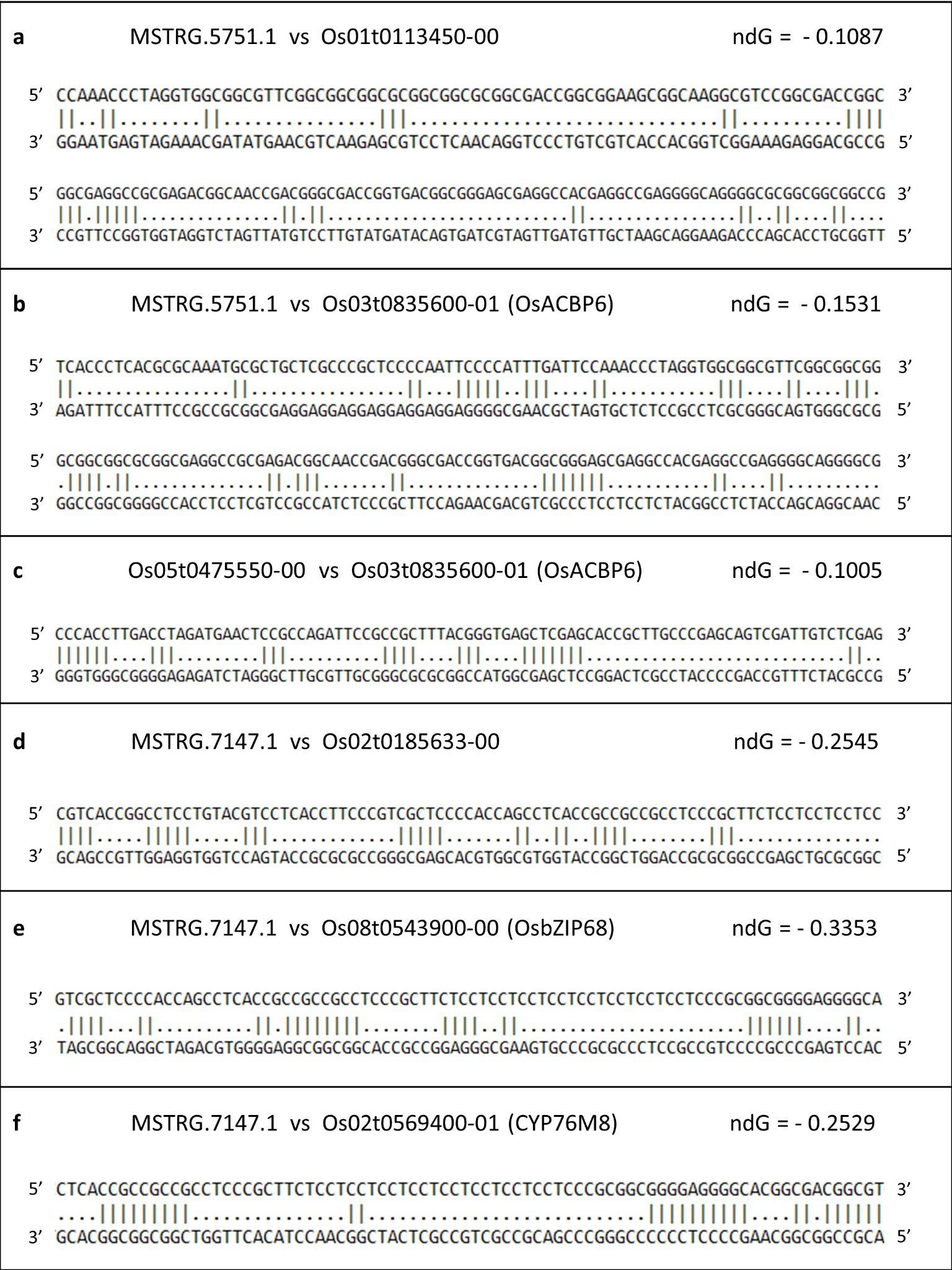


## Supplementary Figure S3. Representative sequence fragments of RNA-RNA interaction between DElncRNAs and their *trans* or *cis* targets predicted by LncTar [[1]](https://www.zotero.org/google-docs/?CxzHue). The normalized delta G (ndG) value of lower than -0.1 was considered a stable complementarily binding. **a**. MSTRG.7147.1 VS Os02t0185633-00. **b**. MSTRG.7147.1 VS Os08t0543900-00. **c**. MSTRG.7167.4 VS Os11t0544100-00. **d**. MSTRG.4754.1 VS Os10t0370800-01. **e**. MSTRG.4676.1 VS Os10t0361000-01.


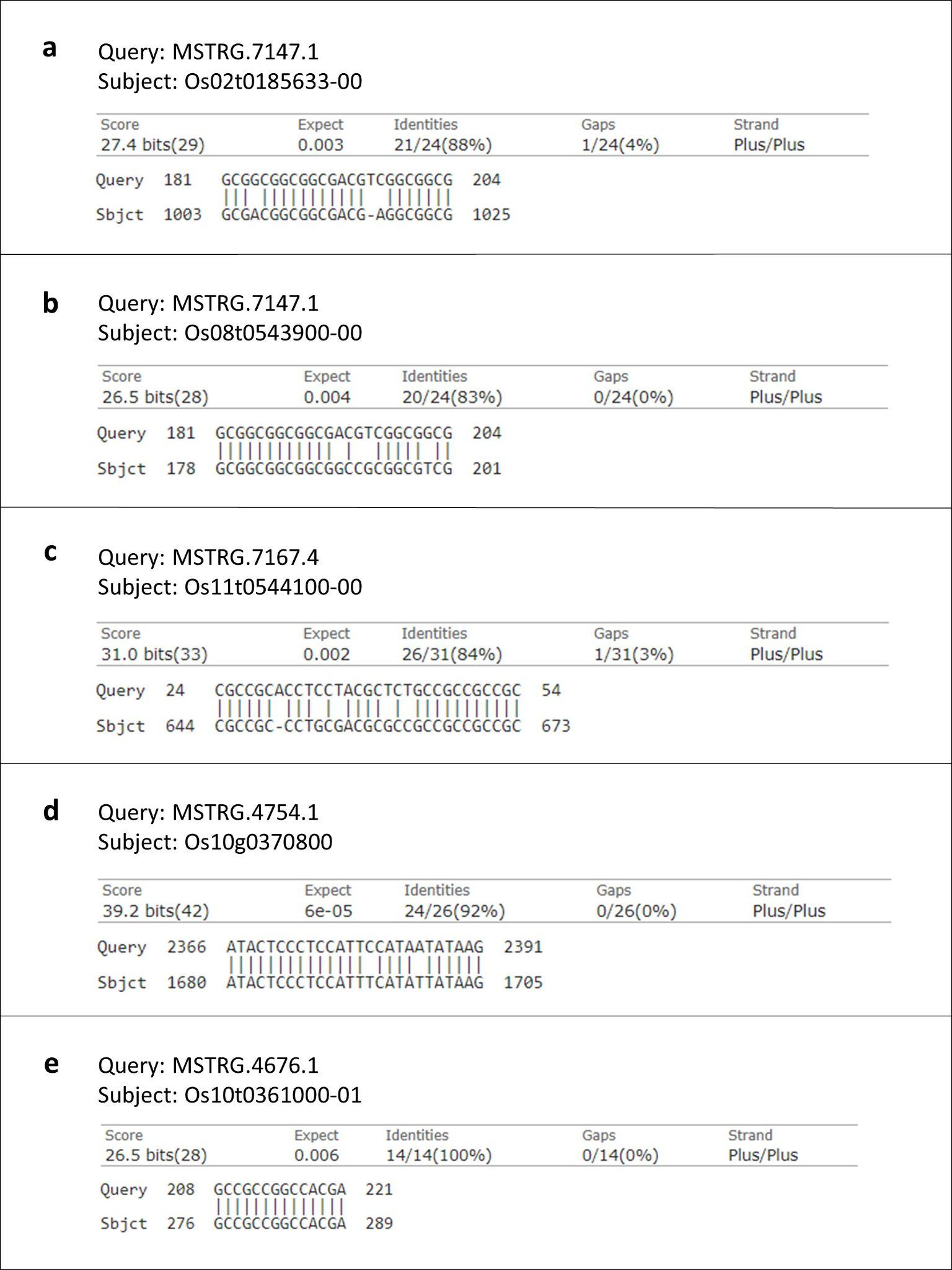


## Supplementary Figure S4. Alignment results between DElncRNAs and their *trans* or *cis* targets. **a**. MSTRG.7147.1 VS Os02t0185633-00. **b**. MSTRG.7147.1 VS Os08t0543900-00. **c**. MSTRG.7167.4 VS Os11t0544100-00. **d**. MSTRG.4754.1 VS Os10t0370800-01. **e**. MSTRG.4676.1 VS Os10t0361000-01.


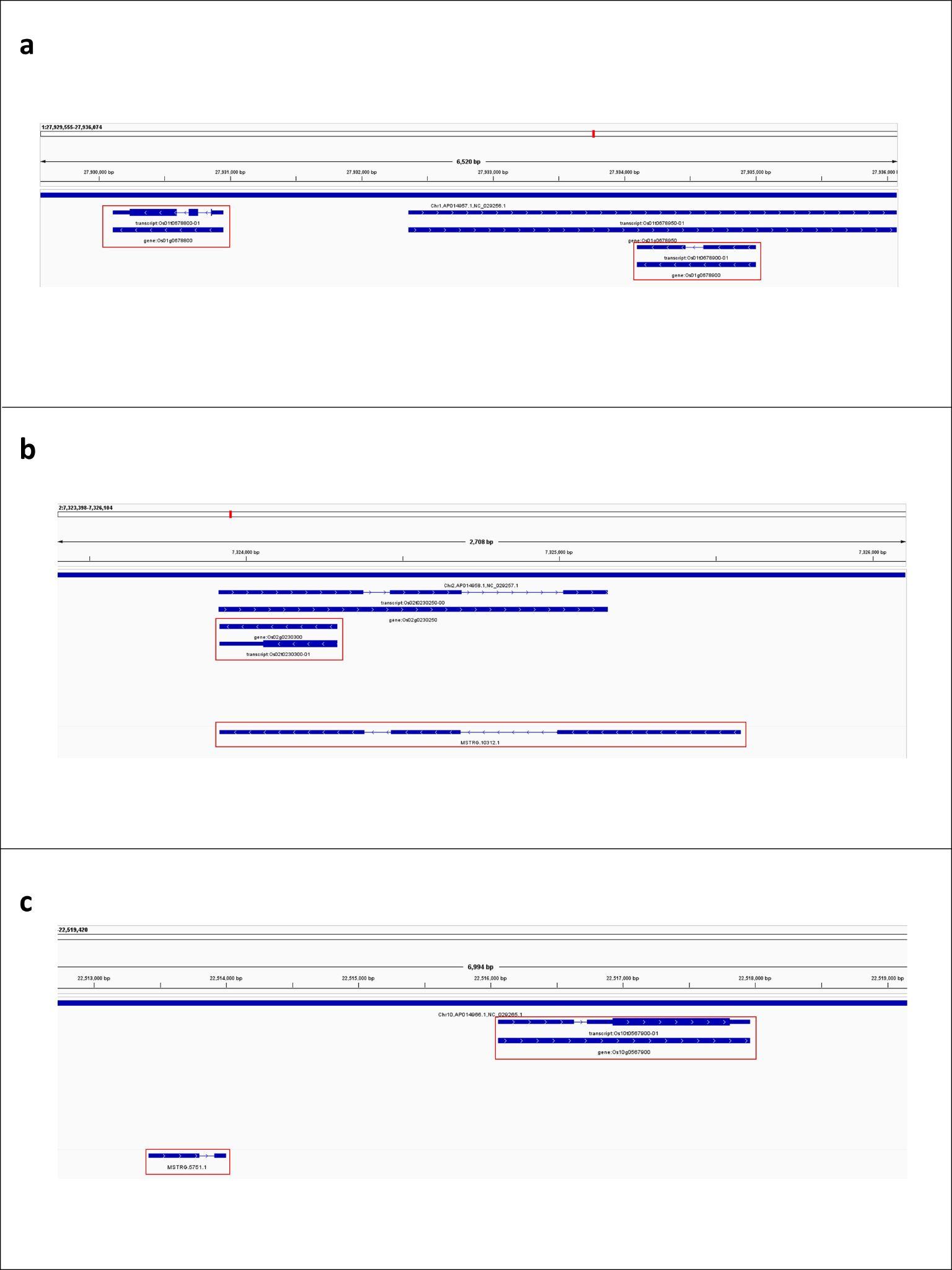


## Supplementary Figure S5. The genomic coordinates of DElncRNAs and their *cis* targets in integrative genomics viewer (IGV) [[2]](https://www.zotero.org/google-docs/?Th5tPd). **a**. Os01t0678900-01 and its targets Os01t0678800-01 (HIPP49). **b**. MSTRG.10312.1 and its target Os02t0230300-01. **c**. MSTRG.5751.1 and its target Os10t0567900-01.

## Supplementary Table S1. The summary of the RNA-Seq and small RNA-Seq data. The clean reads are the sequencing reads passed the filtering steps. Q30(%) indicates the rate of clean reads with at least 99.9% accuracy. The mapped reads are the number of clean reads mapped to the reference genome.

| Rice line | | WT | | OsRpp30-OE | | OsRpp30-KO | |
| --- | --- | --- | --- | --- | --- | --- | --- |
| Sample ID | | WT01 | WT02 | OE01 | OE02 | KO01 | KO02 |
| RNA-Seq | Clean Reads | 71,408,346 | 83,381,550 | 67,967,616 | 69,543,422 | 70,534,266 | 69,706,594 |
|  | Q30 (%) | 94.77 | 94.37 | 94.76 | 94.52 | 94.32 | 94.23 |
|  | Mapped Reads | 67,327,582 (94.28%) | 77,927,847 (93.45%) | 64,188,399 (94.43%) | 65,466,643 (94.13%) | 66,667,542 (94.51%) | 65,893,194 (94.52%) |
| Small RNA-Seq | Clean Reads | 18,446,588 | 12,167,100 | 12,674,116 | 12,418,685 | 14,601,287 | 16,899,916 |
|  | Q30 (%) | 98.61 | 98.45 | 98.58 | 98.58 | 98.59 | 98.45 |
|  | Mapped Reads | 16,931,702 (91.79%) | 11,148,875 (91.63%) | 11,688,504 (92.22%) | 11,459,505 (92.28%) | 13,467,786 (92.24%) | 15,491,217 (91.66%) |

## Supplementary Table S2. The predicted novel lncRNAs and their highly similar lncRNAs in NONCODE, CANTATAdb, and RiceLncPedia databases (identity >= 90%, length coverage >= 90%). The prefixes of the names of the lncRNAs we predicted, NONCODE, CANTATAdb, and RiceLncPeida are ‘MSTRG’, ‘NONOSA’, ‘CNT’, and ‘Osa’, respectively.

| Predicted lncRNA | NONCODE | CANTATAdb | RiceLncPedia |
| --- | --- | --- | --- |
| MSTRG.10686.3 | NONOSAT000718 | CNT20188088 | Osa02LNT0160900.1 |
|  | NONOSAT000473 | CNT20186305 | Osa11LNT0093300.1 |
|  |  | CNT20187678 | Osa12LNT0565900.1 |
|  |  |  | Osa04LNT0485100.1 |
| MSTRG.12637.1 | NONOSAT000134 | CNT20186861 | Osa02LNT0190100.1 |
|  |  |  | Osa06LNT0500400.1 |
|  |  |  | Osa01LNT0008700.1 |
|  |  |  | Osa11LNT0089200.1 |
| MSTRG.13742.1 | NONOSAT000657 | CNT20187819 | Osa03LNT0463200.1 |
| MSTRG.16892.1 | NONOSAT000340 | CNT20188167 | Osa12LNT0125900.1 |
|  |  | CNT20186630 |  |
|  |  | CNT20187333 |  |
|  |  | CNT20188101 |  |
|  |  | CNT20186686 |  |
|  |  | CNT20188015 |  |
|  |  | CNT20186136 |  |
| MSTRG.19604.1 | NONOSAT000340 | CNT20188167 | Osa12LNT0125900.1 |
|  |  | CNT20186630 |  |
|  |  | CNT20187333 |  |
|  |  | CNT20188101 |  |
|  |  | CNT20186686 |  |
|  |  | CNT20188015 |  |
|  |  | CNT20186136 |  |
| MSTRG.24992.1 | NONOSAT000352 | CNT20188690 | Osa12LNT0127700.1 |
| MSTRG.25244.1 | NONOSAT000340 | CNT20187333 | Osa12LNT0125900.1 |
|  |  | CNT20186630 |  |
|  |  | CNT20188167 |  |
|  |  | CNT20188101 |  |
|  |  | CNT20186686 |  |
|  |  | CNT20188015 |  |
|  |  | CNT20186136 |  |
| MSTRG.8373.1 | NONOSAT000065 | CNT20186542 | Osa12LNT0125800.1 |
|  | NONOSAT001035 | CNT20187077 |  |
|  | NONOSAT000771 | CNT20187387 |  |
|  | NONOSAT000678 | CNT20188317 |  |
|  | NONOSAT000802 |  |  |

## Supplementary Table S3. The summary of the genomic coordinates and expression level of DElncRNA-DEmRNA *trans*-targeting pairs. A total of four DElncRNAs and their *trans* DEmRNA targets are listed sequentially.

| No. | DElncRNA | *Trans* DEmRNA target | Chromosome | Strand | Position | Up or Down- Regulation (OsRpp30-OE/OsRpp30-KO) | Log2Ratio  (OsRpp30-OE/OsRpp30-KO) | P-adjusted value |
| --- | --- | --- | --- | --- | --- | --- | --- | --- |
| 1 | MSTRG.5751.1 |  | 10 | forward | 22,513,417 - 22,514,001 | down | -9.56 | 9.72 x 10^-7^ |
|  |  | Os01t0113450-00 | 1 | forward | 731,489 - 733,719 | down | -11 | 3.9 x 10^-9^ |
|  |  | Os07t0153150-02 | 7 | reverse | 2,806,942 - 2,809,736 | down | -10.31 | 4.24 x 10^-8^ |
|  |  | Os03t0835600-01 | 3 | reverse | 35,105,148 - 35,112,495 | down | -12.02 | 1.73 x 10^-11^ |
| 2 | MSTRG.4506.1 |  | 10 | forward | 5,804,437 - 5,805,533 | down | -10.41 | 1.9 x 10^-8^ |
|  |  | Os06t0684000-02 | 6 | reverse | 28,504,190 - 28,508,808 | down | -13.02 | 4.62 x 10^-14^ |
|  |  | Os03t0835600-01 | 3 | reverse | 35,105,148 - 35,112,495 | down | -12.02 | 1.73 x 10^-11^ |
| 3 | Os05t0475550-00 |  | 5 | forward | 23,374,087 - 23,377,731 | down | -11.09 | 1.14 x 10^-9^ |
|  |  | Os07t0153150-02 | 7 | reverse | 2,806,942 - 2,809,736 | down | -10.31 | 4.24 x 10^-8^ |
|  |  | Os03t0835600-01 | 3 | reverse | 35,105,148 - 35,112,495 | down | -12.02 | 1.73 x 10^-11^ |
| 4 | MSTRG.7147.1 |  | 11 | reverse | 19,534,498 - 19,534,758 | up | 6.12 | 1.68 x 10^-2^ |
|  |  | Os08t0543900-00 | 8 | forward | 27,237,307 - 27,241,279 | up | 10.35 | 1.48 x 10^-7^ |
|  |  | Os02t0569400-01 | 2 | forward | 21,690,378 - 21,692,113 | up | 10.79 | 1.68 x 10^-8^ |
|  |  | Os02t0185633-00 | 2 | reverse | 4,774,362 - 4,776,220 | up | 6.29 | 1.41 x 10^-2^ |

## Supplementary Table S4. The summary of the genomic coordinates and expression level of DElncRNA-DEmRNA *cis*-targeting pairs. A total of 21 DElncRNAs and their *cis* DEmRNA targets are listed sequentially.

| No. | DElncRNA | *Cis* DEmRNA target | Chromosome | Strand | Position | Up or Down- Regulation (OsRpp30-OE/OsRpp30-KO) | Log2Ratio  (OsRpp30-OE/OsRpp30-KO) | P-adjusted value |
| --- | --- | --- | --- | --- | --- | --- | --- | --- |
| 1 | MSTRG.3207.1 |  | 1 | reverse | 35,110,199 - 35,111,309 | down | -1.36 | 1.04 x 10^-3^ |
|  |  | Os01t0823600-01 | 1 | forward | 35,175,501 - 35,176,458 | up | 1.01 | 3.47 x 10^-6^ |
|  |  | Os01t0823700-01 | 1 | reverse | 35,176,451 - 35,180,214 | down | -2.52 | 6.66 x 10^-21^ |
| 2 | MSTRG.10312.1 |  | 2 | reverse | 7,323,916 - 7,325,579 | down | -1.06 | 8.35 x 10^-3^ |
|  |  | Os02t0230300-01 | 2 | reverse | 7,323,916 - 7,324,292 | down | -1.12 | 1.71 x 10^-2^ |
|  |  | Os02t0229900-00 | 2 | forward | 7,310,363 - 7,310,992 | up | 3.85 | 7.32 x 10^-3^ |
| 3 | MSTRG.17424.2 |  | 4 | forward | 20,338,833 - 20,341,183 | down | -1 | 1.36 x 10^-2^ |
|  |  | Os04t0410400-01 | 4 | forward | 20,280,360 - 20,281,976 | down | -2.94 | 9.91 x 10^-15^ |
|  |  | Os04t0412300-02 | 4 | forward | 20,373,145 - 20,377,754 | up | 1.44 | 6.5 x 10^-4^ |
| 4 | Os02t0306600-01 |  | 2 | reverse | 12,009,034 - 12,009,419 | down | -4.4 | 1.51 x 10^-5^ |
|  |  | Os02t0306550-00 | 2 | reverse | 12,008,815 - 12,015,423 | down | -6.84 | 5.1 x 10^-4^ |
|  |  | Os02t0307800-02 | 2 | forward | 12,086,040 - 12,089,186 | up | 1.31 | 2.88 x 10^-2^ |
| 5 | Os01t0678900-01 |  | 1 | reverse | 27,934,099 - 27,935,001 | down | -1.16 | 2.67 x 10^-2^ |
|  |  | Os01t0678800-01 | 1 | reverse | 27,930,111 - 27,930,950 | down | -1.33 | 4.91 x 10^-2^ |
|  |  | Os01t0678000-00 | 1 | forward | 27,875,639 - 27,878,341 | down | -1.42 | 5.14 x 10^-6^ |
| 6 | MSTRG.4760.1 |  | 10 | reverse | 11,783,794 - 11,788,322 | up | 1.30 | 1.10 x 10^-3^ |
|  |  | Os10t0370800-01 | 10 | reverse | 11,698,993 - 11,713,558 | down | -2.22 | 1.78 x 10^-3^ |
| 7 | MSTRG.4754.1 |  | 10 | forward | 11,750,575 - 11,754,139 | down | -2.45 | 1.13 x 10^-5^ |
|  |  | Os10t0370800-01 | 10 | reverse | 11,698,993 - 11,713,558 | down | -2.22 | 1.78 x 10^-3^ |
| 8 | MSTRG.5939.1 |  | 11 | reverse | 685,589 - 691,785 | down | -3.58 | 4.9 x 10^-15^ |
|  |  | Os11t0114700-01 | 11 | reverse | 664,534 - 671,701 | up | 3.41 | 1.4 x 10^-3^ |
|  |  | Os11t0114700-03 | 11 | reverse | 665,262 - 671,668 | down | -3.66 | 1.23 x 10^-4^ |
| 9 | Os06t0128200-03 |  | 6 | reverse | 1,485,977 - 1,486,358 | up | 1.01 | 5.76 x 10^-6^ |
|  |  | Os06t0129900-01 | 6 | reverse | 1,581,991 - 1,585,738 | down | -1.19 | 2.08 x 10^-2^ |
| 10 | MSTRG.9888.1 |  | 2 | forward | 3,655,000 - 3,656,464 | up | 8.02 | 4.74 x 10^-3^ |
|  |  | Os02t0167300-02 | 2 | forward | 3,621,165 - 3,622,794 | down | -5.23 | 2.77 x 10^-25^ |
| 11 | MSTRG.24195.1 |  | 7 | reverse | 2,028,789 - 2,031,923 | down | -2.63 | 4.38 x 10^-3^ |
|  |  | Os07t0141100-01 | 7 | reverse | 2,128,467 - 2,130,049 | down | -1.22 | 3.95 x 10^-3^ |
| 12 | MSTRG.26195.1 |  | 7 | forward | 29,591,347 - 29,591,875 | up | 1.67 | 2.98 x 10^-2^ |
|  |  | Os07t0694500-01 | 7 | forward | 29,576,587 - 29,578,832 | up | 1.26 | 1.07 x 10^-19^ |
| 13 | MSTRG.5751.1 |  | 10 | forward | 22,513,417 - 22,514,001 | down | -9.56 | 9.72 x 10^-7^ |
|  |  | Os10t0567900-01 | 10 | forward | 22,516,057 - 22,517,963 | down | -1.96 | 6.32 x 10^-3^ |
| 14 | Os12t0136500-01 |  | 12 | reverse | 1,772,560 - 1,774,555 | down | -1.67 | 3.23 x 10^-2^ |
|  |  | Os12t0137700-01 | 12 | reverse | 1,830,784 - 1,832,039 | down | -1.33 |  |
| 15 | Os01t0924500-01 |  | 1 | forward | 40,479,136 - 40,479,456 | up | 6.16 | 3.35 x 10^-3^ |
|  |  | Os01t0924966-00 | 1 | reverse | 40,526,591 - 40,527,179 | down |  | 2.2 x 10^-3^ |
| 16 | MSTRG.7167.4 |  | 11 | forward | 19,911,133 - 19,913,708 | down | -1.22 | 1.39 x 10^-2^ |
|  |  | Os11t0544100-00 | 11 | reverse | 19,988,741 - 19,991,058 | up | 1.10 | 2.03 x 10^-3^ |
| 17 | Os05t0378900-03 |  | 5 | reverse | 18,319,191 - 18,319,432 | down | -3.94 | 3.81 x 10^-8^ |
|  |  | Os05t0378800-02 | 5 | reverse | 18,313,449 - 18,318,436 | down | -1.91 | 1.86 x 10^-2^ |
| 18 | MSTRG.4676.1 |  | 10 | forward | 11,079,933 - 11,087,253 | up | 5.93 | 6.12 x 10^-4^ |
|  |  | Os10t0361000-01 | 10 | forward | 11,110,251 - 11,110,999 | down | -1.01 | 2.25 x 10^-4^ |
| 19 | Os02t0661500-01 |  | 2 | forward | 26,784,533 - 26,788,011 | up | 1.42 | 5.28 x 10^-3^ |
|  |  | Os02t0661900-01 | 2 | forward | 26,799,675 - 26,802,249 | down | -1.08 | 8.59 x 10^-3^ |
| 20 | Os03t0178600-01 |  | 3 | reverse | 4,144,020 - 4,144,383 | up | 1.71 | 4.5 x 10^-2^ |
|  |  | Os03t0180500-01 | 3 | reverse | 4,217,189 - 4,219,053 | up | 1.89 | 6.54 x 10^-4^ |
| 21 | Os03t0268000-03 |  | 3 | forward | 8,887,710 - 8,889,633 | up | 1.76 | 1.33 x 10^-2^ |
|  |  | Os03t0267300-02 | 3 | reverse | 8,841,311 - 8,843,013 | up | 1.29 | 2.41 x 10^-22^ |

## Supplementary Table S5. The summary of the genomic coordinates and expression level of the RNAs in the lncRNA-miRNA-mRNA competing endogenous RNA (ceRNA) network. The DEmiRNAs and their DElncRNAs/DEmRNAs targets are listed sequentially.

| DEmiRNA | DEmiRNA Target | Category of RNA | Chromosome | Strand | Position | Up or Down- Regulation (OsRpp30-OE/OsRpp30-KO) | Log2Ratio  (OsRpp30-OE/OsRpp30-KO) | P-adjusted value |
| --- | --- | --- | --- | --- | --- | --- | --- | --- |
| osa-miR166k-3p  osa-miR166l-3p | MSTRG.24461.2 | lncRNA | 7 | forward | 4,984,672 - 4,994,785 | up | 1.04 | 7.83 x 10^-6^ |
|  | Os12t0281300-02 | mRNA | 12 | reverse | 10,606,359 - 10,611,914 | up | 1.35 | 1.92 x 10^-6^ |
|  | Os02t0274700-01 | mRNA | 2 | forward | 10,054,334 - 10,056,434 | up | 3.15 | 3.95 x 10^-5^ |
|  | Os02t0273000-01 | mRNA | 2 | reverse | 9,928,936 - 9,937,374 | up | 1.42 | 3.4 x 10^-4^ |
|  | Os05t0189425-00 | mRNA | 5 | reverse | 5,488,413 - 5,490,689 | up | 1.44 | 1.44 x 10^-4^ |
|  | Os04t0629700-02 | mRNA | 4 | reverse | 32,033,925 – 32,038,725 | up | 1.79 | 1.07 x 10^-3^ |
| osa-miR1882a  osa-miR1882b  osa-miR1882c  osa-miR1882d  osa-miR1882e-5p  osa-miR1882f  osa-miR1882g  osa-miR1882h | MSTRG.24461.2 | lncRNA | 7 | forward | 4,984,672 - 4,994,785 | up | 1.04 | 7.83 x 10^-6^ |
|  | Os03t0170200-01 | mRNA | 3 | reverse | 3,760,652 - 3,762,362 | up | 1.22 | 1.08 x 10^-6^ |
|  | Os01t0961600-02 | mRNA | 1 | reverse | 42,395,716 - 42,401,147 | up | 2.63 | 5.57 x 10^-3^ |
|  | Os06t0230100-00 | mRNA | 6 | forward | 6,757,996 - 6,762,787 | up | 1.66 | 2.71 x 10^-2^ |
|  | Os04t0398800-01 | mRNA | 4 | reverse | 19,695,374 - 19,709,946 | up | 1.16 | 3.72 x 10^-6^ |
|  | Os01t0823600-01 | mRNA | 1 | forward | 35,175,501 - 35,176,458 | up | 1.01 | 3.47 x 10^-6^ |
|  | Os10t0345701-01 | mRNA | 10 | forward | 10,341,313 - 10,342,904 | up | 2.36 | 1.95 x 10^-4^ |
|  | Os11t0568800-00 | mRNA | 11 | reverse | 21,213,554 - 21,216,313 | up | 1.06 | 2.8 x 10^-2^ |
|  | Os09t0469400-01 | mRNA | 9 | reverse | 17,857,352 - 17,868,668 | up | 1.20 | 9.13 x 10^-3^ |
|  | Os05t0355133-01 | mRNA | 5 | forward | 16,830,126 - 16,840,001 | up | 1.19 | 9.51 x 10^-4^ |
|  | Os03t0596900-03 | mRNA | 3 | reverse | 22,219,267 - 22,222,046 | up | 1.08 | 5.94 x 10^-5^ |
|  | Os02t0491400-01 | mRNA | 2 | reverse | 17,156,517 - 17,160,895 | up | 1.05 | 3.84 x 10^-13^ |
| osa-miR171d-5p | MSTRG.16012.6 | lncRNA | 3 | reverse | 31,997,920 - 32,000,674 | up | 1.50 | 4.6 x 10^-2^ |
|  | Os03t0660400-00 | mRNA | 3 | forward | 25,893,155 - 25,893,521 | up | 2.93 | 9.17 x 10^-4^ |
|  | Os02t0783700-01 | mRNA | 2 | reverse | 33,260,120 - 33,264,235 | up | 1.24 | 4.76 x 10^-2^ |
|  | Os03t0851400-01 | mRNA | 3 | forward | 35,840,625 - 35,843,776 | up | 1.84 | 1.5 x 10^-2^ |
|  | Os11t0460900-00 | mRNA | 11 | forward | 15,700,955 - 15,701,662 | up | 1.35 | 7.19 x 10^-3^ |
|  | Os04t0322100-01 | mRNA | 4 | reverse | 14,863,727 - 14,864,824 | up | 1.06 | 8.15 x 10^-6^ |
|  | Os03t0232800-01 | mRNA | 3 | reverse | 7,043,271 - 7,046,128 | up | 1.03 | 3.18 x 10^-2^ |
|  | Os05t0518600-01 | mRNA | 5 | forward | 25,714,880 - 25,722,087 | up | 1.97 | 3.32 x 10^-5^ |
| osa-miR1846a-5p  osa-miR1846b-5p | MSTRG.17424.2 | lncRNA | 4 | forward | 20,338,833 - 20,341,183 | down | -1 | 1.36 x 10^-2^ |
|  | Os05t0554000-03 | mRNA | 5 | forward | 27,542,126 - 27,546,073 | down | -2.52 | 3.76 x 10^-3^ |
|  | Os01t0755500-01 | mRNA | 1 | forward | 31,699,518 - 31,701,126 | down | -2.69 | 1.47 x 10^-5^ |
|  | Os04t0107650-00 | mRNA | 4 | reverse | 465,092 - 467,098 | down | -3.69 | 1.47 x 10^-3^ |
|  | Os09t0554200-01 | mRNA | 9 | reverse | 21,956,407 - 21,957,337 | down | -1.54 | 2.16 x 10^-14^ |
|  | Os11t0186900-01 | mRNA | 11 | reverse | 4,415,933 - 4,418,435 | down | -1.51 | 1.27 x 10^-3^ |
|  | Os08t0127100-02 | mRNA | 8 | forward | 1,561,827 - 1,566,957 | down | -1.26 | 5.02 x 10^-8^ |
|  | Os07t0684800-02 | mRNA | 7 | reverse | 29,040,452 - 29,042,127 | down | -1.24 | 1.59 x 10^-2^ |
|  | Os06t0495800-01 | mRNA | 6 | forward | 17,284,744 - 17,286,325 | down | -4.08 | 1.17 x 10^-4^ |
|  | Os05t0582600-01 | mRNA | 5 | forward | 29,000,212 - 29,002,044 | down | -1.17 | 1.99 x 10^-2^ |
|  | Os01t0595600-01 | mRNA | 1 | forward | 23,343,306 - 23,345,362 | down | -2.01 | 1.39 x 10^-2^ |
|  | Os05t0482400-01 | mRNA | 5 | forward | 23,728,568 - 23,738,372 | down | -1.19 | 2.86 x 10^-2^ |
|  | Os08t0378800-02 | mRNA | 8 | reverse | 17,814,500 - 17,817,588 | down | -1.22 | 2.31 x 10^-2^ |
|  | Os03t0302200-01 | mRNA | 3 | forward | 10,651,199 - 10,655,589 | down | -2.76 | 1.56 x 10^-4^ |
|  | Os12t0193100-02 | mRNA | 12 | reverse | 4,755,625 - 4,760,787 | down | -1.08 | 3.99 x 10^-7^ |
|  | Os04t0438200-01 | mRNA | 4 | reverse | 21,791,510 - 21,792,300 | down | -4.42 | 8.84 x 10^-6^ |
|  | Os01t0370000-01 | mRNA | 1 | reverse | 15,192,860 - 15,195,695 | down | -1.92 | 5.17 x 10^-3^ |
|  | Os02t0242100-01 | mRNA | 2 | reverse | 8,033,812 - 8,035,457 | down | -2.16 | 5.42 x 10^-3^ |
|  | Os03t0253600-02 | mRNA | 3 | reverse | 8,107,052 - 8,108,260 | down | -1.12 | 6.09 x 10^-3^ |
|  | Os09t0572100-02 | mRNA | 9 | reverse | 22,852,331 - 22,854,317 | down | -11.6 | 1.17 x 10^-11^ |
|  | Os01t0101600-02 | mRNA | 1 | forward | 72,823 - 77,699 | down | -1.87 | 2.06 x 10^-2^ |
|  | Os10t0579800-01 | mRNA | 10 | forward | 23,128,998 - 23,131,238 | down | -1.17 | 4.32 x 10^-2^ |
|  | Os01t0236400-01 | mRNA | 1 | forward | 7,557,338 - 7,560,496 | down | -2.96 | 6.32 x 10^-3^ |
|  | Os08t0203350-01 | mRNA | 8 | reverse | 6,006,493 – 6,007,052 | down | -4.08 | 9.4 x 10^-6^ |

##

##

##

## Supplementary Table S6. The representative DEmiRNA-DElncRNA targeting pairs predicted by psRNATarget [[3]](https://www.zotero.org/google-docs/?S33d55).


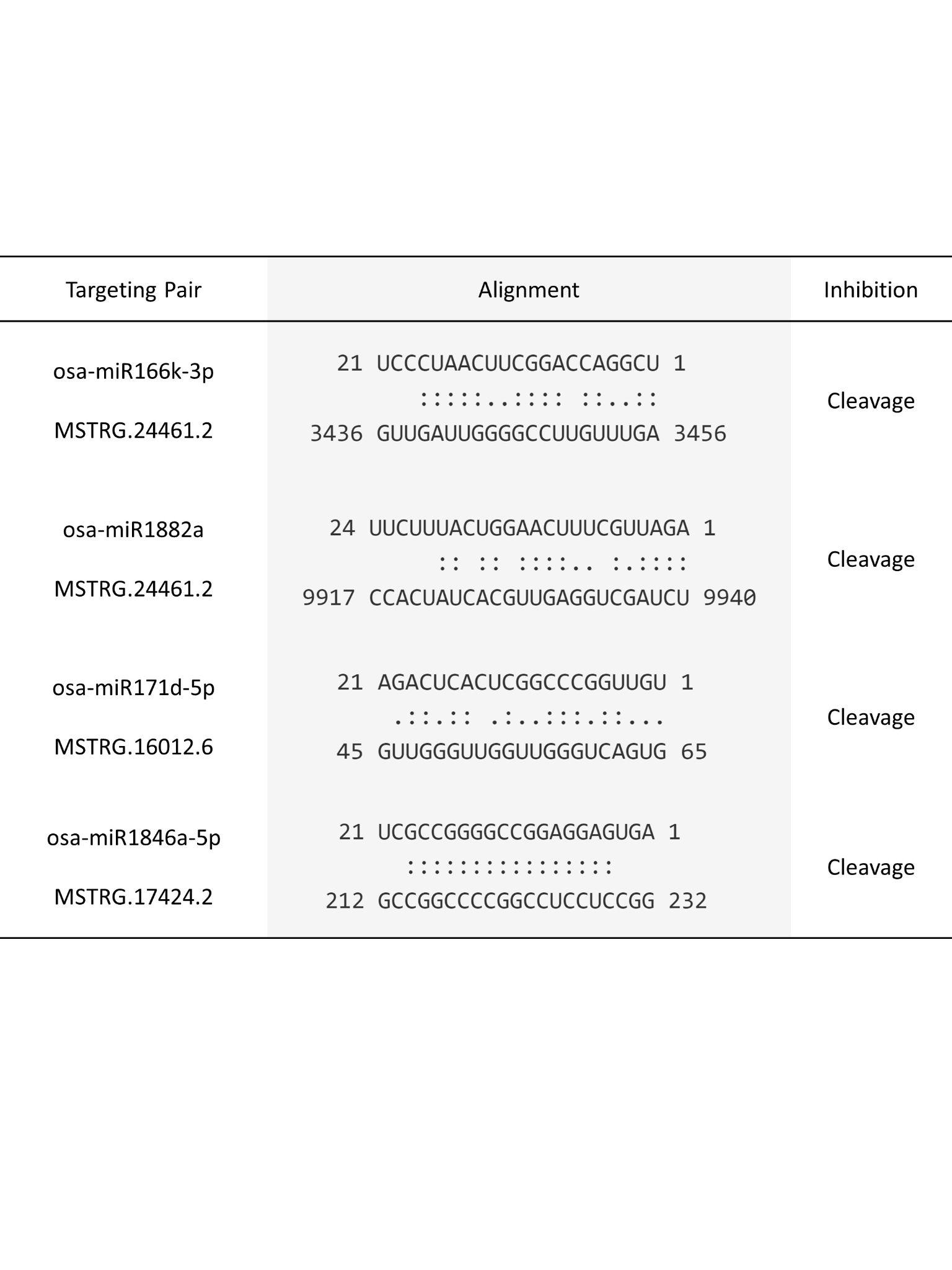


## Supplementary Table S7. The representative DEmiRNA-DEmRNA targeting pairs predicted by psRNATarget.


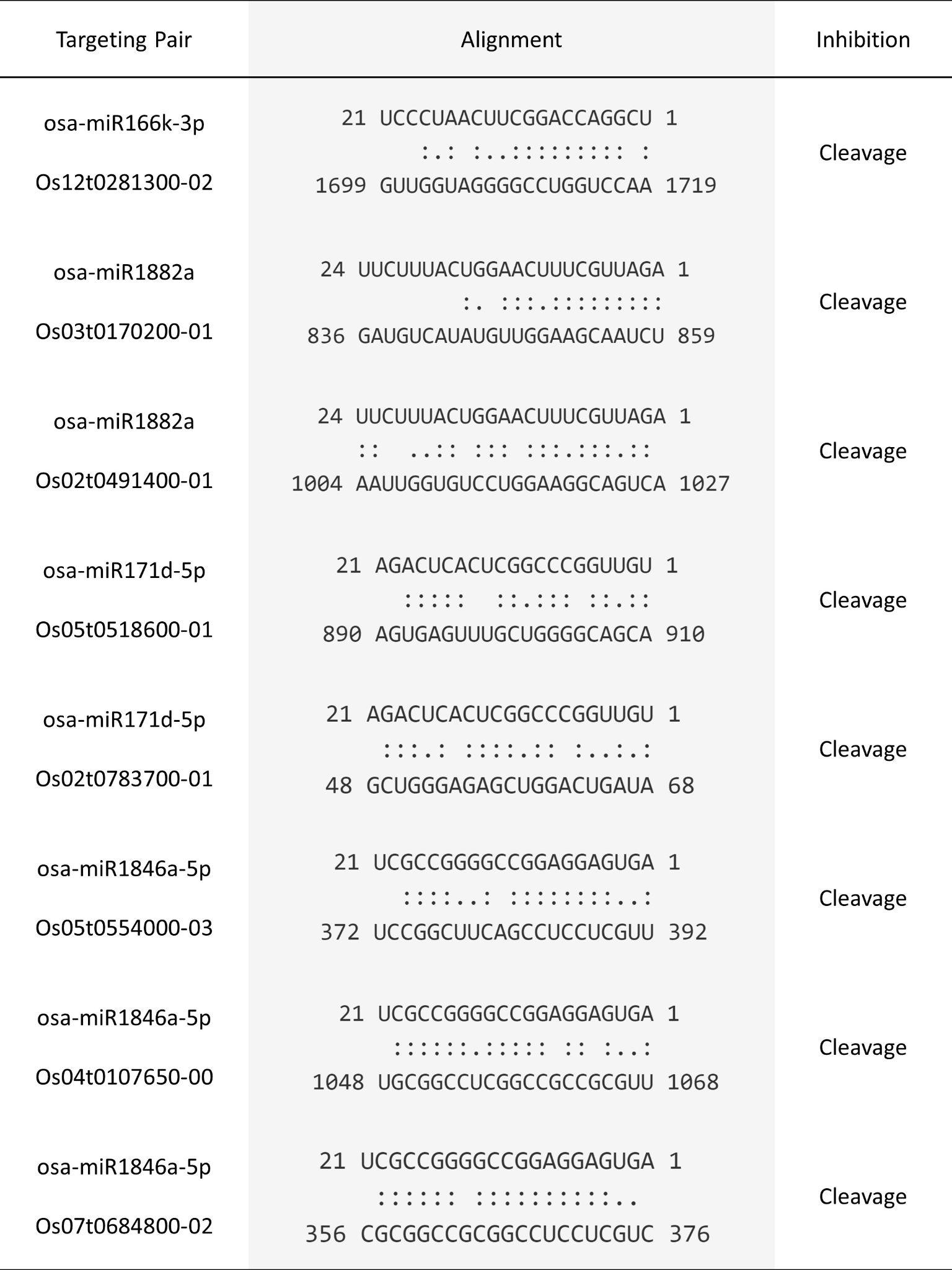


References

[1. Li J, Ma W, Zeng P, Wang J, Geng B, Yang J, et al. LncTar: a tool for predicting the RNA targets of long noncoding RNAs. Brief Bioinform. 2015;16:806–12.](https://www.zotero.org/google-docs/?P64OWP)

[2. Thorvaldsdóttir H, Robinson JT, Mesirov JP. Integrative Genomics Viewer (IGV): high-performance genomics data visualization and exploration. Brief Bioinform. 2013;14:178–92.](https://www.zotero.org/google-docs/?P64OWP)

[3. Dai X, Zhao PX. psRNATarget: a plant small RNA target analysis server. Nucleic Acids Res. 2011;39 Web Server issue:W155–9.](https://www.zotero.org/google-docs/?P64OWP)
